# Genome-wide analysis of the human malaria parasite *Plasmodium falciparum* transcription factor PfNF-YB shows interaction with a CCAAT motif

**DOI:** 10.18632/oncotarget.23053

**Published:** 2017-12-09

**Authors:** Wânia Rezende Lima, David Correa Martins, Kleber Simônio Parreira, Pedro Scarpelli, Miriam Santos de Moraes, Pantelis Topalis, Ronaldo Fumio Hashimoto, Célia R. S. Garcia

**Affiliations:** ^1^ Departamento de Fisiologia, Instituto de Biociências, Universidade de São Paulo, São Paulo, Brazil; ^2^ Centro de Matemática, Computação e Cognição, Universidade Federal do ABC, Santo André, Brazil; ^3^ Departamento de Bioquímica, Instituto de Química, Universidade de São Paulo, São Paulo, Brazil; ^4^ Institute of Molecular Biology and Biotechnology, FORTH, Hellas, Greece; ^5^ Departamento de Ciência da Computação, Instituto de Matemática e Estatística, Universidade de São Paulo, São Paulo, Brazil; ^*^ Instituto de Ciências Exatas e Naturais-Medicina, Universidade Federal de Mato Grosso-Campus Rondonópolis, Mato Grosso, Brazil

**Keywords:** transcription factor, CCAAT-box, malaria, Plasmodium falciparum, signaling

## Abstract

Little is known about transcription factor regulation during the *Plasmodium falciparum* intraerythrocytic cycle. In order to elucidate the role of the *P. falciparum* (Pf)NF-YB transcription factor we searched for target genes in the entire genome. PfNF-YB mRNA is highly expressed in late trophozoite and schizont stages relative to the ring stage. In order to determine the candidate genes bound by PfNF-YB a ChIP-on-chip assay was carried out and 297 genes were identified. Ninety nine percent of PfNF-YB binding was to putative promoter regions of protein coding genes of which only 16% comprise proteins of known function. Interestingly, our data reveal that PfNF-YB binding is not exclusively to a canonical CCAAT box motif. PfNF-YB binds to genes coding for proteins implicated in a range of different biological functions, such as replication protein A large subunit (DNA replication), hypoxanthine phosphoribosyltransferase (nucleic acid metabolism) and multidrug resistance protein 2 (intracellular transport).

## INTRODUCTION

*Plasmodium falciparum* merozoites invade red blood cells (RBC), and over 48 hours develop and multiply through the ring, trophozoite and schizont stages [1, 2]. This asexual erythrocytic stage of the life cycle is attractive to study because it is responsible for the symptoms of the disease, and can be cultivated *in vitro*. The genome sequence has contributed enormously to our understanding of molecular and cellular aspects of the life cycle [3], however gene regulation in *P. falciparum* is still poorly understood.

Few transcription factors or regulatory motifs have been identified and characterized in malaria parasites [4-6]. Gene expression analysis revealed that transcription is generally monocistronic and finely regulated [7-12]. The AT-rich *P. falciparum* genome and the complex life cycle involving differential gene expression at morphologically distinct stages make it difficult to predict and to characterize transcription factors. Analysing the genome revealed a relative paucity of transcription-associated proteins and specific cis-regulator motifs [13], but the regulatory machinery includes a canonical TATA-box binding protein and RNA polymerase II-dependent messenger RNA production [14, 15].

Using bioinformatics, including genomic and proteomic tools, some orthologues of general transcription factors such as the TFIIB family [15] have been predicted. Genes encoding transcriptional regulators such as the Myb1 protein [16], high mobility group box (HMGB) proteins, and Apetala2 (AP2) [17] have been characterized in *P. falciparum*, and recently a new set of 129 cis-regulatory elements was reported [18].

Our group has studied for more than a decade how melatonin, a tryptophan-derived metabolite, modulates the cycle of *P. falciparum* and *P. chabaudi* parasites [19, 20]. A signaling cascade is induced by the indolamine, thus prompting a rise in the second messengers IP_3_, Ca^2+^ and cyclic AMP [21-23]. cAMP activates protein kinase A (PKA), and consequently modulates anion conductance and vesicle trafficking in *P. falciparum [24-26]*. A variety of regulatory factors are modulated by second messenger signaling in vertebrates [27-30]. It is a feature of cell biology that Ca^2+^ has a role regulating long-term cell adaptation by controlling gene expression (for review see [31]).

On PlasmoDB database (http://www.plasmodb.org), Pf11_0477 (now described as PF3D7_1146600) transcript was predicted to have around 3906 bp according to northern blotting results [32]. Moreover, in the rodent malaria parasites *P. berghei,* has been identified a Pf Nf-YB orthologue named ORP1 (Oocyst Rupture Protein 1) also containing an histone-fold domain (HFD) NF_YB-like. The authors found that mutant parasites in which orp1 gene has been deleted, oocyst rupture, normally occurring in mosquito midgut around day 12 post blood meal, is inhibited but growth of asexual stages was not affected [33]. The rupture of the oocyst is an essential step for parasite transmission and ORP1 plays a fundamental role in this mechanism.

We have demonstrated that melatonin induces posttranslational modification of the PfNF-YB protein by increasing its ubiquitination, allowing PfNF-YB abundance to be controlled by the ubiquitin-proteasome system [34]. In transcriptional studies PfNF-YB was detected in asexual schizont and in sexual gametocyte stages [14, 35]. The NF-Y protein complex binds a CCAAT-box and in *P. falciparum* is comprised of three subunits A (Pf13_0043), B and C (Pf14_0374) [14]. In other organisms the NF-Y subunit B recognises and binds to the CCAAT sequence in the promoter region of various genes [36-38]. For example, NF-YB binds to mammalian genes during the G_2_/M phase of the cell cycle and regulates the expression of topoisomerase IIα, cyclin B1, CDC25C, E2F, CDC2 and thymidine kinase [39-41]. In contrast, in *P. falciparum*, the target gene(s) bound by PfNF-YB during the parasite cycle life have not been identified, and the role of this transcription factor is unknown. Our group reported that a cAMP analogue (6-Bnz-cAMP) modulates PfNF-YB transcript and proteins levels, raising the possibility of a role for this protein in parasite signaling pathways [34].

In this research, we identified several DNA binding sites candidates for PfNF-YB transcription factor at the schizont stage. Our results suggest that PfNF-YB is involved in the control of numerous different biological functions in the parasite, including protein translation folding and modification, intracellular transport, nucleic acid metabolism and cell redox homeostasis.

## RESULTS

### PfNF-YB is expressed at higher levels in mature forms of the intraerythrocytic parasite

**Figure 1:**
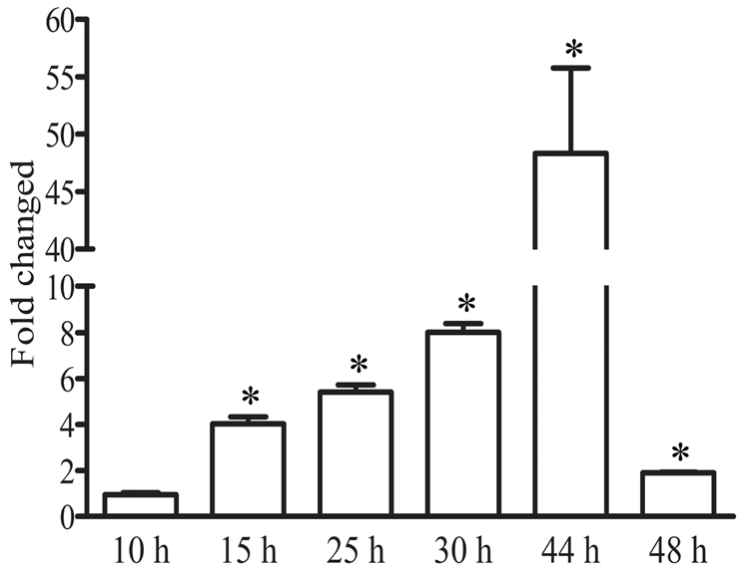
PfNF-YB expression throughout the intraerythrocytic cycle of *P. falciparum* Samples of a synchronized parasite population were collected at various time points (10-48 h) after invasion. RT-PCR analysis of PfNF-YB mRNA level reveals differential expression during the intraerythrocytic cycle. Statistical analysis was performed by *t* test (p<0.05). The amount of mRNA in each sample was normalized by the level of 18S RNA.

In order to investigate PfNF-YB function during the *P. falciparum* asexual stage we followed its transcript expression throughout the intraerythrocytic developmental cycle using a synchronized parasite population. Figure 1 shows the RT-PCR results after analysing the transcripts at 5 different time-points of development up to 48-hours post invasion (hpi). The analysis revealed that PfNF-YB transcript abundance peaks at 44 hpi followed by a drastic drop in abundance at 48 hpi. Following invasion, the PfNF-YB transcript level is low at early ring stage (10 hpi) and slowly increases during parasite maturation. Our quantitative PCR results are similar in pattern to those in the microarray database available at http://plasmodb.org and published for *P. falciparum* 3D7 strain [42]. The higher expression of PfNF-YB in mature parasites led us to postulate that the protein might regulate genes involved in DNA replication and cell division.

### The PfNF-YB transcription factor binds to *P. falciparum* DNA sequences

In various different eukaryotic cells NF-Y proteins are involved in cell cycle control, regulating several genes [40, 41]. We have shown here that *P. falciparum* NF-YB is highly expressed in late stages and located at least in part in the nucleus of both trophozoite and schizont forms. During these developmental stages, DNA duplication, followed by nuclear division and cytokinesis to produce new merozoites is essential to allow successful parasite multiplication and invasion of new red blood cells. Therefore, we asked whether PfNF-YB at the schizont stage (44 hpi) can bind to DNA and regulate expression of target genes involved in the parasite cell cycle. To answer this question, a genome-wide PfNF-YB chromatin immunoprecipitation (ChIP-on-chip) assay was performed using a high density whole genome tiling array (Roche NimbleGen Inc) and material purified from schizonts. Chromatin immunoprecipitation was done using PfNF-YB-specific rabbit antibody attached to beads, and, as controls, non-specific rabbit IgG attached to beads and beads alone. Before performing the ChiP-on-chip assay, qPCR with selected genes (PFI1665w and Pf14_0489) was used to test whether an enrichment of promoter regions was obtained using the PfNF-YB antibody. Using this approach, we observed that the putative promoter region of both PFI1665w and Pf14_0489 was enriched in the fraction bound to the PfNF-YB-specific antibody compare to the fraction bound to nonspecific rabbit IgG or beads alone (Supplementary Figure 1). For the more extensive ChiP-on-chip experiment, to determine the target genes for PfNF-YB binding we selected target sequences using three criteria: 1) the minimum Log 2 ratio value was set at 0.5; 2) the maximum false discovery rate (FDR) value was set at 0.095 (0.095 % FDR cut-off); and 3) probes covering more than 150 bp sequence were required (at least 4 probes, each probe of 50 bp). In the analysis we took into account the potential presence of putative promoter regions in the first exon and in other intragenic regions, since some genes in *P. falciparum* such as the *var* gene family have a promoter within the gene [43-45].

**Figure 2:**
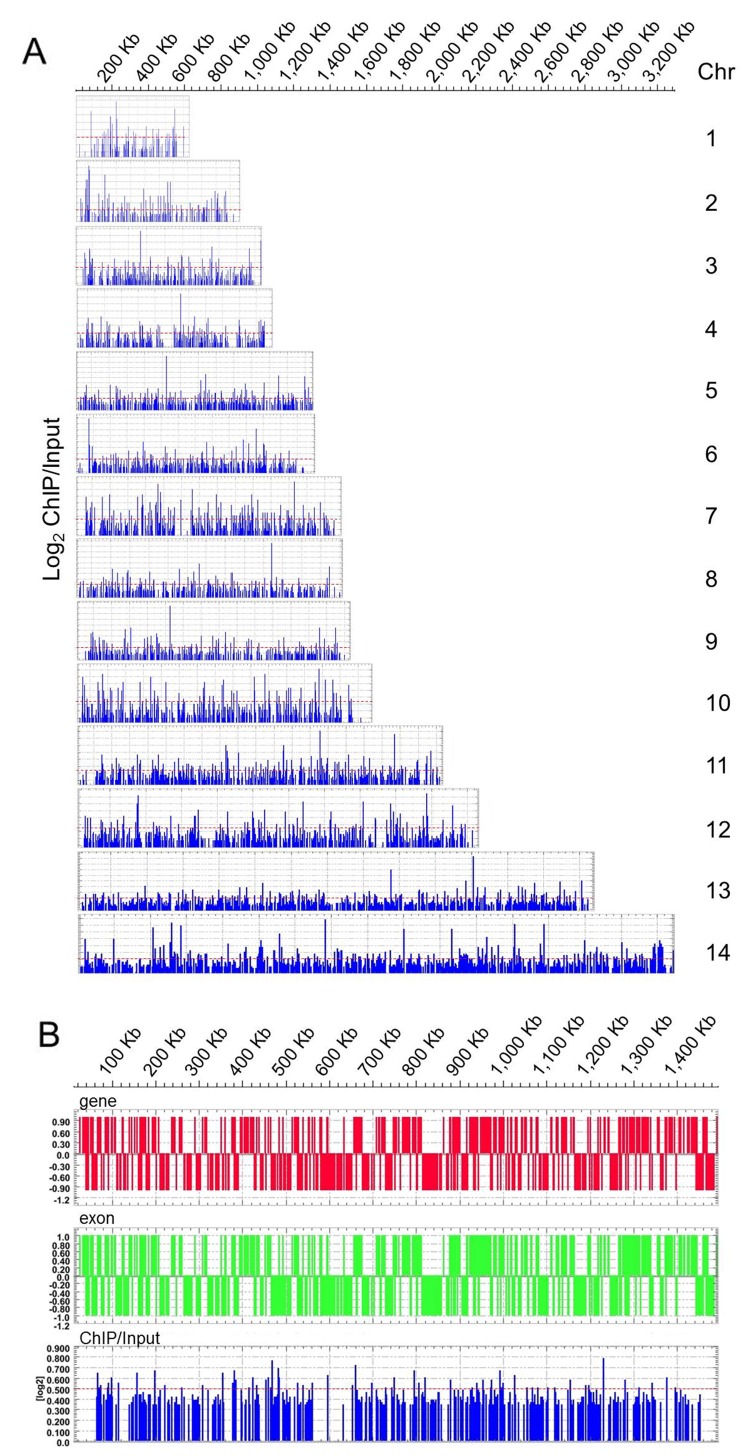
Genome-wide PfNF-YB occupancy by high-density ChIP-on-chip assay The display (Deva) shows the chromosomal map position and location of DNA enrichment due to PfNF-YB binding. **(A)** The blue panel peaks shows the ChiP/Input ratio for all 14 chromosome in schizont stage 3D7 parasites. PfNF-YB occupancy for each gene was calculated as the average of log_2_ ratios of hybridization values for immunoprecipitated and input chromatin. **(B)**. PfNF-YB occupancy on chromosome 7. Schematic display (Deva) of the blue peaks on chromosome 7 shows PfNF-YB occupancy in the gene region (red bars) and the exon region (green bars).The candidate promoter regions were estimated from false discovery rate (FDR) values (0.095) and by Log2 value ratio ≥ 0.5 (dotted red line). The ratio data were randomized 20 times to evaluate the probability of “false positives”. Each peak was assigned an FDR score based on the randomization. Chromosome numbers are indicated on the right, chromosomal position (Kb) on top.

The graphic display generated by the Deva software shows PfNF-YB binding sites distributed in several putative promoter regions of target genes (Figure 2) throughout the 14 chromosomes of the *P. falciparum* genome (Figure 2A). PfNF-YB binding was homogeneously distributed on all chromosomes. A higher resolution display of ChiP-on-chip results for PfNF-YB binding to Chromosome7 is shown in Figure 2B. The candidate PfNF-YB target genes were selected by an enrichment above Log_2_ ≥ 0.5, a threshold that is represented in the figure by a dotted red line (Figure 2A and 2B). We exemplify these results using the MAL7P1.25 and PFB0325c target genes in Supplementary Figure 2. The Deva program established the peaks by searching for 4 or more probes whose signals were above the specified cutoff values, ranging from 90% to 15%, using a 500 bp sliding window. The cutoff values were a percentage of a hypothetical maximum, which was the mean + 6 standard deviations. The ratio data were then randomized 20 times to evaluate the probability of “false positives”. Each peak was then assigned a false discovery rate (FDR) score based on the randomization. We observed that the positive peaks for MAL7P1.25 and PFB0325c (Supplementary Figure 2) do not possess a high log2 ratio value and this may be due to the high AT content of the *P. falciparum* genome. AT-rich prokaryotic genomes may have less precise control of transcription initiation [46, 47], a phenomenon which may explain the low signal to noise ratio in our ChIP-on-chiP data. Inevitably this background will decrease the ease of definition of specific peaks, but it does not preclude the specificity of antibody binding to the transcription factor and of the transcription factor binding to target genes. As shown in Supplementary Table 1 we noted 297 target genes bound by PfNF-YB that fulfilled all three selection criteria.

### Validation of target genes

**Figure 3:**
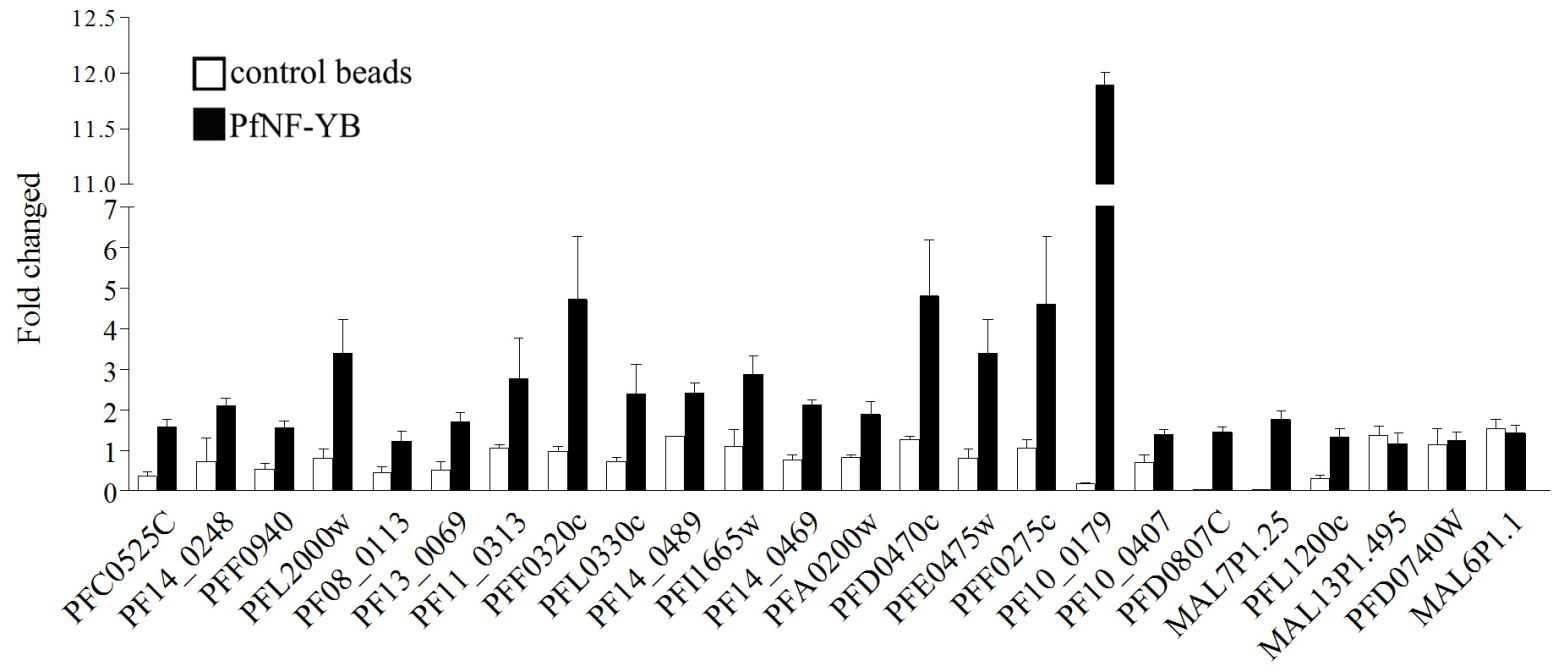
ChIP-on-chip validation Target genes were selected randomly to perform ChIP/qPCR from schizont stage parasites. Twenty-one different genes showed a PfNF-YB enrichment of at least 2-fold compare to control beads. The last three genes were selected as negative binding targets for PfNF-YB. Input samples were used to normalize the relative values. The control beads were used as a negative control for the ChIP assay. These analyses were derived from three independent assays. The primers used in the analyses can be found in Supplementary Table 3. Gene accession numbers (www.PlasmoDB.org) are indicated at the bottom of the graphic.

Signal enrichment and FDR values are criteria that suggest binding regions, but in order to properly assess the array results, it was crucial to validate the targets identified from the ChIP-on-chip assay. Therefore, we used 3 different ChIP assays from schizonts (44 hpi) to confirm the enrichment and to measure the sensitivity and specificity of the detection by qPCR (Figure 3). Potential PfNF-YB targets were selected according to their enrichment, FDR value and biological function. We also chose three different regions with poor enrichment as controls in the qPCR using ChIP DNA. The analysis demonstrated that the selected 21 genes were positively enriched as judged by qPCR following immunoprecipitation with the PfNF-YB-specific antibody beads compare with the control immunoprecipitations. The three control genes MAL13P1.495, PFD0740w and MAL6P1.1 that were not enriched did not show any difference by qPCR between the PfNF-YB antibody and control bead immunoprecipitations. The qPCR method was shown to be more sensitive to detect enrichment than the ChIP-on-chip approach, once candidates had been identified.

### PfNF-YB targets are involved in several biological processes in *P. falciparum*

**Figure 4:**
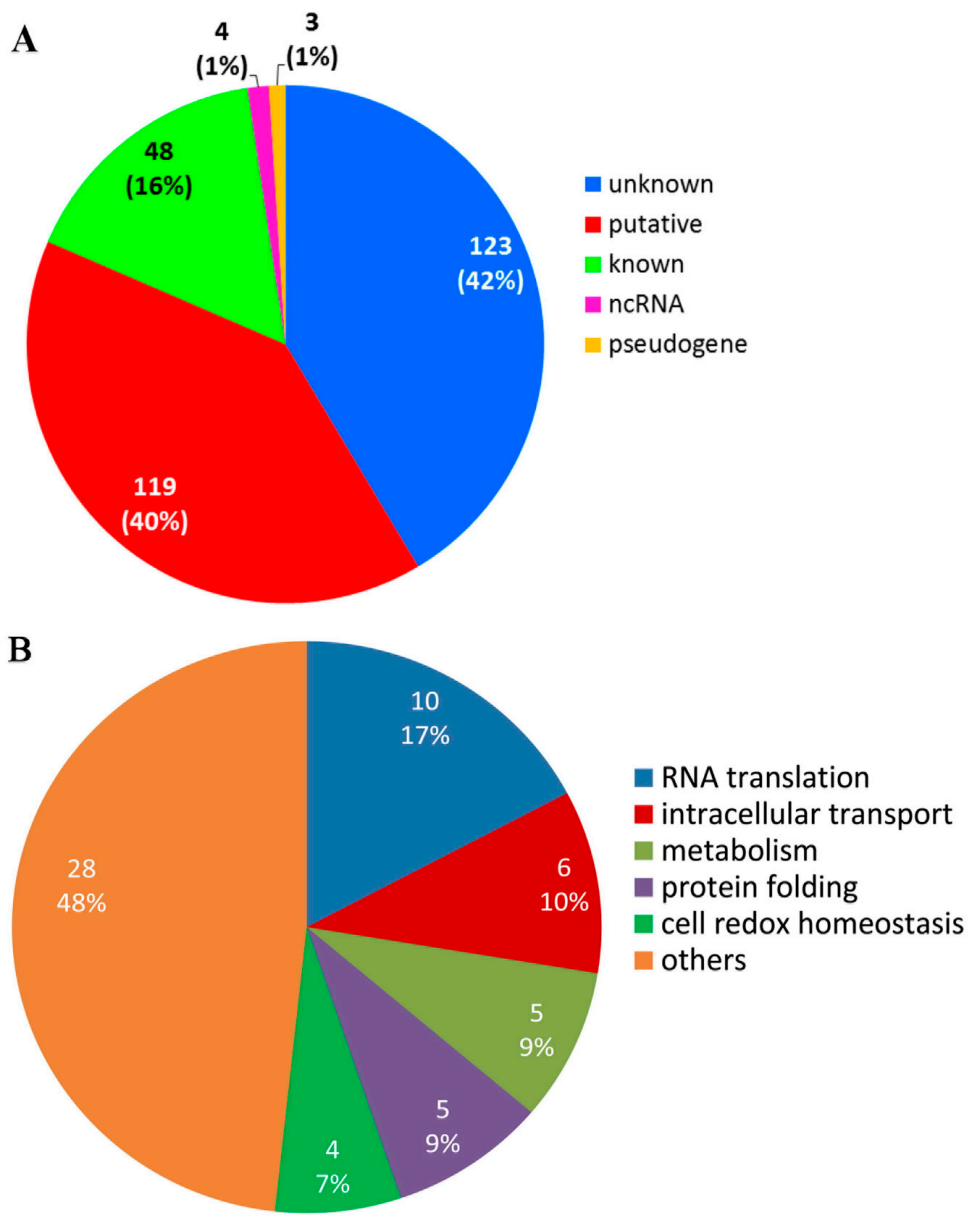
PfNF-YB binds to different gene functions in *P. falciparum* **(A)** PfNF-YB target genes classified by RNA type and protein function. Genes coding for proteins with unknown function (42%) represent the biggest group, followed by the groups represented by the genes with putative (40%) and known (16%) function. **(B)** Identification of biological processes associated to the target genes bound by PfNF-YB. A group containing 167 coding and putative genes that have a biological process attribute according to the Gene Ontology section from PlasmoDB database were plotted. Fifty-two per cent of these genes are involved in RNA translation, intracellular transport, metabolism, protein folding and cell redox homeostasis.

Analysing the list of identified target genes, we classified and inferred the biological function of the proteins coded by genes bound by PfNF-YB transcription factor using the annotation provided by the PlasmoDB database (version 7.0). From 297 targets identified by the ChIP-on-chip experiment, 42% are of unknown function, 40% are proteins with putative function, 16% are of known function, 1% are pseudogenes and 1% are non-coding RNA (Figure 4A). With respect to the group of 167 proteins of known and putative function associated with a biological process (a gene ontology term), we observed a high diversity of function (Figure 4B). The major groups include proteins involved in RNA translation, protein folding, intracellular transport, cell redox homeostasis and metabolism (for more detail see Supplementary Table 2).

### PfNF-YB recognizes the CCAAT motif in the parasite genome

**Figure 5:**
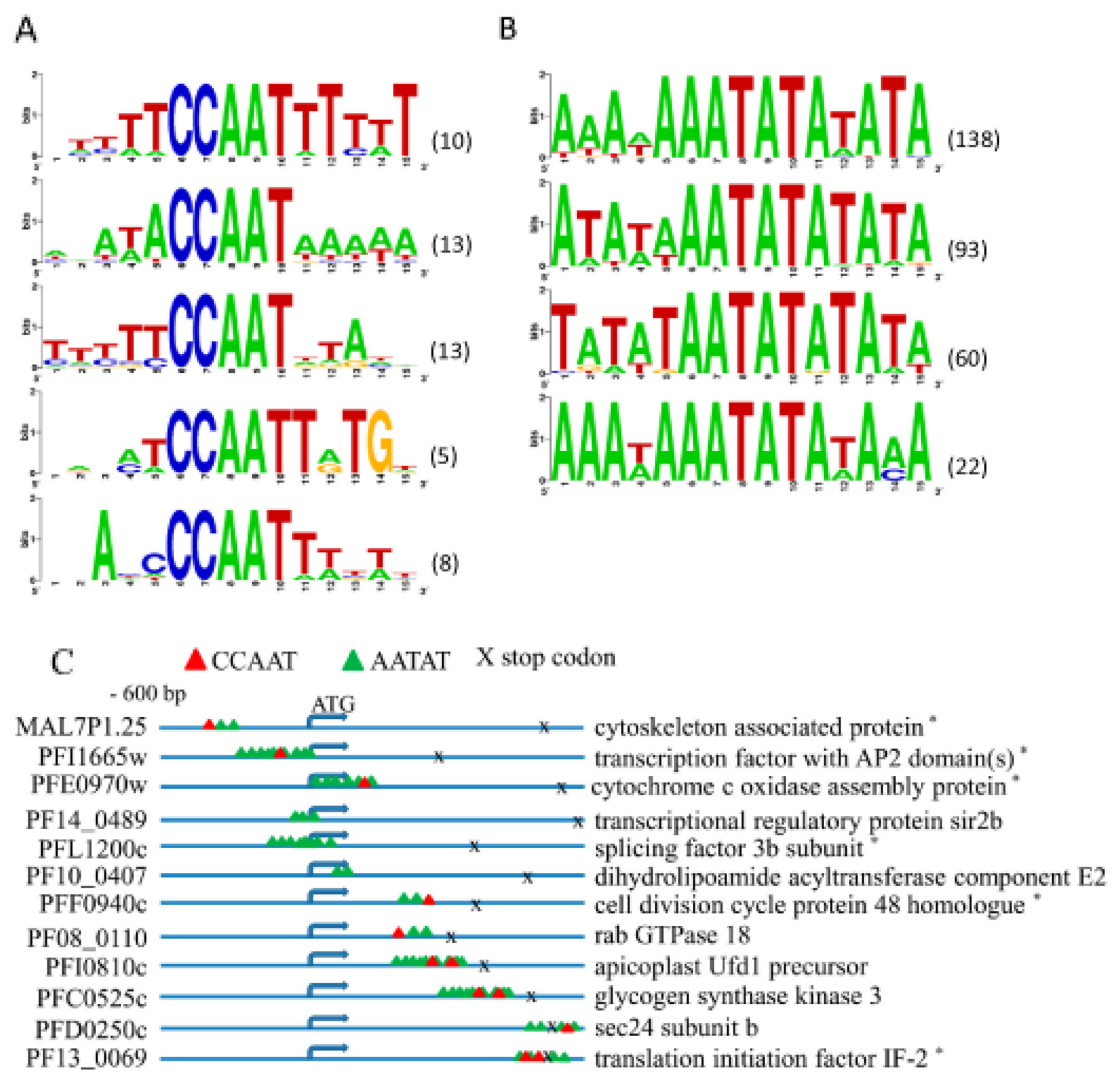
Identification of CCAAT and AATAT motifs bound by PfNF-YB transcription factor **(A)** The CCAAT motif sequence recognized by PfNF-YB was found in the promoter regions of 140 genes. Forty-five out of these 140 regions containing 49 CCAAT motifs were investigated for putative consensus sequences. **(B)** AATAT motif sequence was found in the promoter regions of 297 genes recognized by PfNF-YB. One hundred and twenty-three of these 297 regions containing 313 AATAT motifs were investigated for putative consensus sequences. The probe sequences covering the target genes were selected and assembled, and the putative promoter sequences were analysed by motif finder WebLogo [67]. The graphic shows the relative entropy-based logo of the detected motif. Values in parenthesis represent the number of gene promoters belonging to the consensus motifs. **(C)** The PfNF-YB target genes present a diversity of binding site regions. The scheme shows the matches to the CCAAT motif and to the putative TATA-box motif. The location of these motifs is both upstream and downstream of the ATG start codon. (*) putative gene.

Transcription factors are crucial in the control of gene expression. Very few transcription factors are known currently in *P. falciparum* (see review [48]), and this scarcity of information has prevented a better identification of transcription factor binding sites. Using bioinformatics tools, we took advantage of the WebLogo program to elucidate motifs in the promoter regions [4, 18, 49, 50]. In vertebrates, the NF-YB protein family binds to the CCAAT motif, but in *P. falciparum* the existence and distribution of this motif has not been investigated. Our aims here were to clarify whether the PfNF-YB recognizes the CCAAT motif in *P. falciparum* and whether or not it binds to others motifs. Probe regions were chosen based on the FDR score and across the range of DNA coverage. Each flanking sequence of the target genes was analysed by ClustalW. First, we searched for the CCAAT motif in both sense and anti-sense strands for the 297 target genes and for the rest of the genome sequence. We found that 157 target genes did not contain the CCAAT motif. Further, we performed Monte Carlo permutation testing [51, 52] in order to determine whether or not the CCAAT motif is over-represented in the putative promoter regions. The simulation took into account the lengths of 297 gene targets identified to obtain a set of new random subsequences from the whole genome, and the number of subsequences that matched at least once with a particular motif was counted. This procedure was repeated 100,000 times to estimate the p-value (i.e., the percentage of repeat executions in which the number of subsequences that contained at least one match equaled or exceeded the actual number of target matches). Indeed, the frequency of the CCAAT motif is significantly higher in the identified PfNF-YB target genes (p-value: 0.00043) than in the rest of the genome. Among these sequences, we found putative promoters of 45 genes containing at least 80% homology in a motif comprising 5 bases upstream and downstream flanking the CCAAT core. We propose 5 consensus sequences for the CCAAT motif in *P. falciparum* according to the WebLogo analyses (Figure 5A). The *P. falciparum* genome is the most AT-rich of all the eukaryotes sequenced to date [51, 52] and this high AT content may explain why the nucleotides flanking the CCAAT motif are more AT-rich compared to those in other eukaryotes where NF-Y binds to CCAAT within a more GC-rich sequence environment. In order to better address this situation we analyzed the proportion of A and T nucleotides flanking the CCAAT motif in the PfNF-YB target genes and compared the distribution to the rest of the genome. We examined 5bp both upstream and downstream of the CCAAT motifs in the genome and found that in target genes the frequency of A(T) is on average 5.32(4.88) matches per CCAAT motif, including CCAAT itself which contains two As (one T), whereas in the rest of the genome, the average frequency of A(T) is 5.66 (4.99) per CCAAT motif and flanking sequence. Analyzing each 15 bp-length subsequence throughout the genome, the average A(T) frequency is 6.05 (6.05) per subsequence. Finally, the average number of A’s or T’s present in the 15 bp subsequences per CCAAT motif in the target genes is 10.2, per CCAAT motif throughout the genome is 10.6, and per 15 bp subsequence throughout the genome is 12.1. These results indicate that the frequencies of A’s and T’s are not higher in nucleotides flanking CCAAT motifs within PfNF-YB target gene sequences compared to CCAAT motifs generally or compared to 15 bp-subsequences in the rest of the genome. Thus, this analysis indicates that the frequency of A’s and T’s flanking the CCAAT motif is a general feature of the parasite genome and not a specific sequence preference dictated by the NF-YB motif in the target genes in *P. falciparum.*

We also searched for other common motifs using the 297 target genes identified by the ChIP-on-Chip approach. Surprisingly, we discovered several motifs that contained an AATAT core with 5 bases upstream and downstream conserved, which were present in the putative promoters of all 297 genes (Figure 5B). As the AATAT sequence is highly conserved in the binding sites for TATA-box protein, we used the TRANSFAC database to evaluate those sequences that matched TATA box binding sites. Taking into account only the selected sequences, and those clusters with at least four highly similar sequences, we detected 83 putative promoters containing non-TATA box motifs. These sequences were analysed by the WebLogo algorithm and 4 consensus AATAT-based motifs were identified, which may be bound by the PfNF-YB transcription factor, or alternatively the consensus motif for the TATA-box in *Plasmodium* may exhibit some sequence diversity (Figure 5B). AATAT-based motifs are abundant in plants and are important to control gene expression in *Arabidopsis* in starvation conditions [53]. This may be a very important finding concerning gene regulation in *P. falciparum.* The position of the AATAT motif and the CCAAT binding sites in representative genes are illustrated in Figure 5C, displaying all the possible positions of CCAAT and AATAT motifs in the PfNF-YB target genes.

### PfNF-YB binds to new motifs

**Figure 6:**
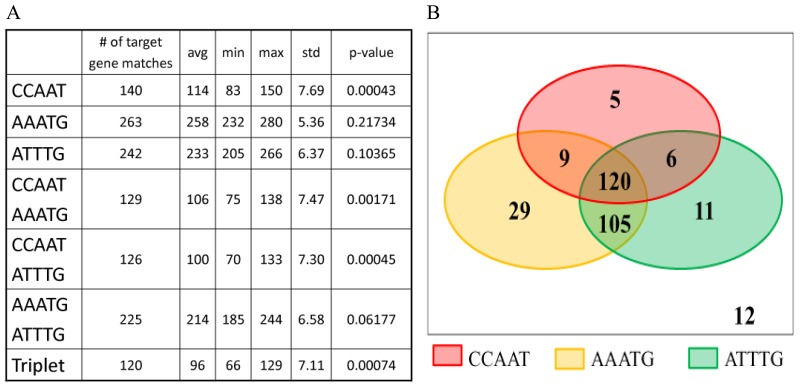
Motif matching in the putative promoter region of PfNF-YB target genes **(A)** The Monte Carlo test was performed taking into account the gene target lengths to obtain new random subsequences. This procedure was executed 100,000 times to estimate the p-value (percentage of executions in which the number of subsequences that matched the considered motifs equalled or exceeded the actual number of target matches). The frequency of the CCAAT motif is significantly higher in predicted genes (p-value: 0.00043) than in the rest of the genome. The three motifs CCAAT, AAATG and ATTTG are also significantly more frequent in the PfNF-YB target genes group compared to the rest of the genome (p-value: 0.00074), which suggests that these motifs are strongly associated. **(B)** A Venn diagram to better illustrate the distribution of the three motifs among the 297 predicted genes. There are 140 matches for CCAAT, 263 matches for AAATG and 242 matches for AATTG. 120 predicted genes (40.4%) match with the three CCAAT, AAATG and ATTTG motifs. Note that only twelve genes (4%) contain none of the three motifs.

In order to establish whether PfNF-YB binds to a new motif in the putative promoter regions we searched for new sequences and compared these sequences with motifs already described [18]. The analysis showed the presence of 263 matches for AAATG (described in [18]) and 242 matches for ATTTG (a new motif) (Figure 6). The pentameric motif AAATG is a part of the ATG(G)AAATG motif found in maize and wheat histone H3 and H4 mRNAs that contain poly(A) sequences at the 3´-end [54]. ATG(G)AAATG analogous sequences have been identified in the plants arabidopsis, alfalfa, rice and wheat [55]. The ATTTG motif (a CCAAT-like motif) was described as binding to NF-YB in pro-a1(V) and collagen (col5a1) promoter regions that lack the CCAAT canonical sequence (or the reversed complement, ATTGG). The analysis has revealed the presence of this motif in *P. falciparum* for the first time. Statistical analysis was performed to determine the permutations of motifs located in the same putative promoter regions. Combining the CCAAT, AAATG and ATTTG motifs, we determined the association between them. In Figure 6 only the associations in which the presence of the motif is not due to a random event are displayed. Furthermore, sequences with associations between the three motifs comprise 120 genes (around 40%) out of the 297 identified PFNF-YB target genes. It is important to note that there are twelve target genes lacking any of the three motifs analysed in this study. These results identified AAATG [18] and revealed a new motif ATTTG as a putative PfNF-YB binding sequence, however, whether PFNF-YB binding is direct or indirect was not addressed here.

## DISCUSSION

Our results showed that mRNA PfNF-YB expression is highest in schizont stages in agreement with data from PlasmoDB, and suggesting that it plays an important role at this multiplicative stage of the asexual blood cycle. Therefore, to explore the PfNF-YB role in the asexual cycle of the parasite using chromatin immunoprecipitation (ChiP) was important to elucidate its binding gene target. ChIP is one of the most powerful tools to elucidate protein-DNA interaction and has been applied to study histone- or transcription factor-DNA interaction in Plasmodium [4, 56-59]. In our results we could observe that the noise was a little bit high, but this can be explained by the fact the NF-Y binding to *cis*-elements containing the highly conserved core sequence CCAAT [60] and rich in the *Plasmodium* genome. Nevertheless, reports suggest that ∼7–8% of human promoters have functional *CCAAT* motifs [61]. Our results showed that 297 genes were found to bind to PfNF-YB. Considering that the *P. falciparum* encodes about 5300 genes, we can say that about 5.6% of putative promoters have motif to PfNF-YB binding in the *P. falciparum* genome. Taking advantage of the *P. falciparum* genome database, we performed ChIP assays combined with a tiling array and identified, for the first time, several target genes bound by the PfNF-YB transcription factor.

Among genes with putative and known function from ChIP-on-chip results and based on the biological function designation available in PlasmoDB database, we found that PfNF-YB target genes play a role in RNA translation, nucleic acid metabolism, protein translation and transport (Figure 5B and Supplementary Table 2). Genes involved in purine nucleotide metabolism are adenosine monophosphate deaminase (MAL13P1.146) and hypoxanthine phosphoribosyltransferase (Pf10_01221). Moreover, PfNF-YB target gene such as replication protein A large subunit (PFD0470c) was described to participate in DNA replication, repair, recombination and checkpoint processes in other eukaryotic cells [62, 63]. The role of NF-Y in nucleic acid metabolism in mammalian cells is clear and well defined [64]. Considering, a) the increase in PfNF-YB mRNA expression during the differentiation of trophozoites to schizonts (Figure 1) when DNA replication begins in the intraerythrocytic parasite suggests a role for PfNF-YB in DNA duplication. Indeed, DNA synthesis is first observed when the average age of parasites was between 30.5 and 32.5 h and then increase logarithmically until the average age of the parasites are between 44 and 48h [64]; b) the nuclear localization of PfNF-YB in these stages [34] ; c) the *in vivo* promoter occupancy by PfNF-YB at schizont form (Supplementary Figure 1); and d) its interaction with genes involved in nucleic acid metabolism (Supplementary Table 2), we can hypothesize that PfNF-YB is implicated in DNA replication and multiplication in schizont stage (44 h).

Protein translation and modification processes are among the most promising areas to target for the development of new anti-malarial drugs. PfNF-YB binds to the genes for several translational proteins, for example translation initiation factor IF-2 (PF13_0069) and peptide release factor 1 (PF14_0265). Moreover, we also identified the eukaryotic translation initiation factor 3 subunit 8 (PFL0310c) and several 40S and 60S ribosomal proteins. The third class of gene that PfNF-YB binds is related to transport. Our results demonstrate that PfNF-YB binds to promoter regions of genes for 15 transporter proteins, among them Sec24b (PFD0250c), Rab GTPase 18 (Pf08_0110) and PfMDR2 (Pf14_0455).

Besides the molecular characterization of the transcription factor, it is very important to know the type of motif it binds. In order to infer *in silico* transcription factor binding sites we applied bioinformatics approaches, such as WebLogo (http://WebLogo.berkeley.edu/). Logos are commonly used for the representation of transcription factor binding site preferences [65]. It also allows us to predict the binding site using the interaction energy with the transcription factor [66, 67]. Therefore, we used WebLogo to determine the motif bound by PfNF-YB in the 297 target genes. The results show that *P. falciparum* NF-YB binds to a variety of motifs. Although the NF-Y transcription factor binds to the cis-element CCAAT motif in most eukaryotic cells, in *P. falciparum* we showed by in silico analysis that PfNF-YB does not bind exclusively to this cis-element, since 157 genes do not possess the CCAAT binding site in the region immunoprecipitated with PfNF-YB antibody. ChIP-on-chip analysis shows the interaction between protein and DNA, but does not address whether this interaction is directed or not. Therefore the 157 target genes lacking a CCAAT binding site may be recognized by PfNF-YB due to interactions with other transcription factors.

Our analysis showed the presence of the pentamer motif AAATG (263 matches, Figure 6), corresponding to the well-known Myc transcription factor binding site, which is found in a number of cold-responsive gene promoters in *Arabidopsis thaliana* [54]. We also detected 242 matches for the ATTTG motif (a CCAAT-like motif), which was described as binding to NF-YB in pro-a1(V) and collagen (col5a1) promoter regions that lack the CCAAT canonical sequence (or the reversed complement, ATTGG), [68]. Based on this information and our results, it appears that the ATTTG motif can replace the CCAAT motif and promote the PfNF-YB binding to DNA in *P. falciparum*. Furthermore, the WebLogo analysis identified the cis-element AATAT (313 matches) that was identified by *in silico* TRANSFAC database search as a TATA-box. The presence of a TATA-box motif corroborates the binding of the PfNF-YB transcription factor to putative promoter regions of target genes. The AATAT motif is also associated with gene signaling in inorganic phosphate starvation in *Arabidopsis* *[53]*. It is not surprising to find *Arabidopsis* cis-element sequences in the *P. falciparum* genome, since the malaria parasite genome is more similar to that of *Arabidopsis* than other non-Apicomplexan taxa [68].

Recently several works have shown the importance of AP2 genes in controlling blood stage and sexual developmental cycle of *Plasmodium*. It has been postulated that ap2-g and ap2-g2 proteins play a role as gene repressors during the life cycle progression of *P. berghei* parasites and they are crucial for gametocytogenesis [69, 70]. By contrast, only non-essential functions were described for ap2-o and ap2-sp proteins in blood stages. Comparing our data with those from Modrzynska and collaborators [69], both for schizonts, we found a total of 50 genes that are potential targets of ap2-g and PfNF-YB proteins (Supplementary Table 4). Out of these 40 (80%) were upregulated after knocking out ap2-g gene in schizont cultures. In addition, the expression of 20 other target genes was also regulated by both ap2-g2 and PfNF-YB proteins. Fifteen (75%) of these genes were upregulated in ap2-g2 knockout parasites. These results suggest that PfNF-YB together with ap2-g or ap2-g2 proteins act as a complex or, at least, separately on different binding sites, to repress the expression of gametocyte genes during the schizont stage. As demonstrated by the authors, gametocytogenesis was lost or significantly reduced in schizonts lacking ap2 or ap2-g2 proteins. Therefore, a hypothetical synergism between ap2-g or ap2-g2 proteins and the PfNF-YB transcription factor, most likely with other regulators, could contribute to these effects on the development of gametocytes in both *P. bergei* and *P. falciparum* species. Examining our data and those recently published by Santos and collaborators (2017) we found that PfAp2-l and PfNF-YB proteins regulate 11 common genes in *P. falciparum* (Supplementary Table 4). PfAp2-l, likewise PfNF-YB, is expressed at the mid to late schizont, and both bind to target genes, such as cAMP-dependent protein kinase catalytic subunit (PKA-c), which was showed to regulate microneme secretion before invasion [71]. ChIP-seq data revealed that PfAp2-l binds the promoter regions of many different histone genes. Indeed, we found that histone H2B is also a common target of PfNF-YB. Moreover, genes encoding proteins involved in RBC remodeling, i. e. early transcribed membrane protein (ETRAMP5), is also a target of PfAp2-l (Santos *et al.*, 2017) and PfNF-YB. This data suggests *that P. falciparum* transcription factors AP2 and NF-YB regulate DNA/ chromatin genes and host-cell remodeling.

Based on target gene function, PfNF-YB can control nucleic acid metabolism, protein translation, transport and regulation of gene expression. However, it is important to highlight the fact that 42% of all target genes identified have no known function. We demonstrate here that PfNF-YB binds to a variety of motifs showing a divergence compared to the situation in most eukaryotic cells, where the CCAAT motif alone is required for NF-YB interaction with a promoter region. In addition, previously we showed that proteasome inhibitor, bortezomibe (BTZ), besides blockage of ring and trophozoite development also decreases PfNF-YB expression [34], leading us to speculate whether PfNF-YB plays a role in intraerythrocytic developmental cycle of parasite. Indeed, both the transcript and protein levels of PfNF-YB increase following the parasite maturation, resulting in the stimulation of PfNf-YB expression in schizont and a decrease in PfNF-YB expression in ring stage [32, 34, 42]. Here, the results of CHIP-on-Chip assay corroborate the hypothesis that PfNF-YB plays a role in DNA replication and parasite division once genes involved with these biological activities were found enriched in this assay. These data suggest that PfNF-YB could be an interesting anti-malarial candidate, which could acts mainly in schizont stage.

## MATERIALS AND METHODS

### Culture of plasmodium falciparum

*P. falciparum* strain 3D7 parasites were cultured in flasks at 37°C and 5% hematocrit in RPMI 1640 medium supplemented with 10% human plasma, and gassed with 90% N_2_, 5% O_2_ and 5% CO_2_ [72]. For synchronization, high parasitemia parasites cultures at the ring stage were centrifuged for 5 min at 800 x g at room temperature, then the supernatant was discarded and the parasitized erythrocytes were incubated for 5 min with 5% sorbitol solution to remove any residual mature parasites. After incubation, the erythrocytes were centrifuged again, washed twice with complete RPMI medium and placed back in culture as previously described [73].

### RNA extraction, cDNA synthesis and real-time PCR

Samples from parasites were harvested at 10, 15, 25, 30, 44 and 48 hr after invasion for qPCR assay. Parasitized erythrocytes were lysed using Trizol LS (Invitrogen) according to the manufacturer’s instructions. cDNA synthesis was performed with 300 ng of random primers and 500 ng of total RNA previously treated with DNAse, using the Superscript II Kit (Invitrogen) as described in the manufacturer’s instructions. Quantitative analysis of the relative levels of specific transcripts in triplicate samples was assessed by SYBER green quantitative real-time PCR (qPCR) in a 7300 Real Time PCR System (ABI) under the following conditions: 50°C for 2 min, 95°C for 10 min, 40 cycles of 95°C for 15 sec; 55°C for 30 sec; 60°C for 30 sec. The efficiency of amplification for each gene was previously determined using dilutions of cDNA or gDNA. Genes outside the 90-110% range were discarded from the analysis. For each reaction 2 to 8 ng of cDNA, oligonucleotides (800 nM final concentration) in Max Mix PCR buffer (ABI) were used. The mRNA was amplified using the following primers. Pf11_0477: 5’-acaagcgaggctagtgacag-3’, 5’-ttcagatattcggttaatggttc-3’; MAL-18S: 5’-aacacaaggaagtttaaggcaacaa-3’, 5’-gcgtgcagcctagttca-3’. The 18S ribosomal RNA gene was amplified as an endogenous control and used to normalize the quantitation results. The reactions were carried out in triplicate and the expression values shown represent the relative amplification from each cDNA sample compared to the control. The significance of the difference between the relative gene expression levels was determined by Student’s *t-test* using three independent assays.

### ChIP-on-chip assay

Schizont stage parasites (44 hour post invasion) were isolated from *P. falciparum* cultures and on three independent occasions were used in chromatin immunoprecipitation assays. Parasites were released from red blood cells by saponin treatment (0.01% saponin, 1x PBS, 1 mM PMSF) as described above, and crosslinking was performed using 2% formaldehyde at 37°C for 10 minutes. Excess formaldehyde was quenched by addition of glycine, with gentle agitation for 5 minutes at room temperature.. The parasites were then lysed in 5 mM HEPES pH 8.0, 85 mM KCl, 0.5% Triton X-100, plus protease inhibitors for 15 minutes. The nuclear fraction was harvested by centrifugation at 3000 rpm and the pellet was incubated with 500 μl of nuclear lysis buffer (50 mM Tris-HCl, 10 mM EDTA pH 8.0, 1% SDS, and protease inhibitors). The DNA was sheared into 200 to 1000 bp fragments by sonication (10 times for 10 seconds using a Sonics Vibra cell Model), and the fractionated chromatin was visualized on an agarose gel stained with ethidium bromide. One milliliter of the supernatant was pre-cleared with 100 μl of protein A-Sepharose beads, by incubation overnight at 4°C with agitation, then the mixture was centrifuged and the pellet discarded. The clarified solution containing fragmented chromatin was incubated overnight at 4°C with 20 μg anti-PfNF-YB or a nonspecific rabbit IgG attached to beads, or with control beads (no antibody attached). The mixture was centrifuged at 300 rpm for 3 minutes and the pellet together with one tenth of the input supernatant were retained. The pellet was washed three times with immunoprecipitation buffer (50 mM HEPES pH 7.5, 500 mM NaCl, 1% Triton X-100, 0.1% sodium deoxycholate, 1 mM EDTA) and then 6 times with wash buffer (10 mM Tris-HCl pH 8.0, 250 mM LiCl, 0.5% NP-40, 0.5% sodium deoxycholate, 1 mM EDTA). Bound material was uncoupled from the Sepharose by reversing the crosslinking at 65°C overnight. The DNA was then purified using the phenol extraction method and dissolved in 30 μl of sterile distilled water. PCR analysis of the DNA immunoprecipitated with PfNF-YB antibody or a control rabbit IgG, and in the beads alone and input fractions was performed as described [74, 75]. The amplicons from PFI1665w (ApiAP2) (5’TCCTGAACGTTCAGGTAAAA3’ and 5’GCAATTCACCTGTTTTCGCT3’), PF14_0489 (Sir2B) (5’TCGTGTCCCGTTAATTTT3’and 5’GTTGATATGCCAGCACCTGA3’) and 18S ribosomal RNA (5’CCACATCTAAGGAAGGCAGC3’ and 5’CACCAGACTTGCCCTCCAA3’) were generated using the following conditions: 95°C for 10 min, 30 cycles of 95°C for 15 sec, 58°C for 30 sec, and 72°C for 45 sec. The 18S ribosomal RNA amplicon served as a loading control [76]. PCR products were resolved by agarose gel electrophoresis and DNA was visualized under UV illumination. The total ChIP DNA was also amplified prior to hybridization with the microarray platform using the Genomeplex WGA kit from Sigma. The quantity and quality of DNA were verified using the Nanodrop and Bioanalyzer apparatus (see below). Amplified dsDNA was labeled with Cy5- (ChIP) or Cy3- (input) and hybridized to a tiling array based on the annotation provided by the PlasmoDB database (version 7:0). The high-density microarray platform of 720,000 features with 3 characters (3 x 720k) covering the whole *P. falciparum* genome was produced by Roche NimbleGen (USA). The ChIP samples were hybridized with this full *P. falciparum* genome and the results were analyzed using the Deva program from Roche. The ChIP-on-chip data were normalized using the Tukey bi-weight function to account for differences between the dyes on the array. Roche NimbleGen scaled the ratios in the.gff files by subtracting the bi-weight mean for the probe log2-ratio values for all features on the array from each log2-ratio value.

### Accession numbers

The raw ChIP-on-chip data presented and discussed in this publication were deposited in NCBI’s Gene Expression Omnibus and are accessible through GEO Series accession number GSE48894 (http://www.ncbi.nlm.nih.gov/geo/query/acc.cgi?acc=GSE48894).

### Bioanalyzer

DNA samples from the chromatin immunoprecipitation assay were dissolved in DNase-free water at a final concentration of 5ng/μl. DNA 7500 lab chips (Agilent Technologies) were loaded with samples as recommended by the manufacturer; the micro channels were filled with 9 μl of gel-dye mix in the appropriate wells and the 5μl marker and the samples in 1μl running buffer were loaded. The samples were gently mixed and then the chips were immediately inserted into the Agilent 2100 Bioanalyzer and processed.

### ChIP/qPCR validation

The validation of the ChIP-on-chip assay was performed by qPCR using specific primers (Supplementary Table 3). Samples of the input material and material bound to either PfNF-YB-specific or non-specific rabbit IgG coated beads and control beads were used at the same molar concentration to verify the IP enrichment compared with the bead-alone control. The qPCR was performed using Power SYBR green mix (Applied Biosystems) in triplicate and from three independent experiments. The relative amount of DNA of target genes was normalized against the input samples and the values of the cycle threshold were calculated for the absolute amount of DNA.

### Motif analysis

To analyze the promoter regions of putative candidate genes, we used *P. falciparum* genome sequences available in the databases: http://plasmodb.org/plasmo/ and http://genome.ucsc.edu/. Sequences upstream of the ATG translation initiation codon, were evaluated as potential promoter regions by analyzing 500 bp upstream and downstream of the PFNF-YB binding site. The WebLogo program was used to represent the DNA sequence of all target genes from the ChIP-on-chip assay.

### Statistical analyses

The statistical significance of differences between means was analysed by Student’s *t* test or Monte Carlo methods, as indicated. The Monte Carlo permutation test [77, 78] was used to determine whether or not the CCAAT, AAATG, ATTTG and AATAT motifs are overrepresented in the putative promoter regions. The simulation took into account the lengths of the 297 gene targets identified. The number of subsequences that matched at least once with a particular motif was counted and the procedure was repeated 100,000 times to derive a p-value.

## SUPPLEMENTARY MATERIALS FIGURES AND TABLES








