# Supplementary material for: Genome-wide analysis of the human malaria parasite Plasmodium falciparum transcription factor PfNF-YB shows interaction with a CCAAT motif
Source: Oncotarget. 2017 Dec 9;8(69):113987–4001. doi: 10.18632/oncotarget.23053 (PMC5768380; doi:10.18632/oncotarget.23053)
Supplement: Supplementary file 1 [file oncotarget-08-113987-s001.pdf]

Genome-wide analysis of the human malaria parasite *Plasmodium falciparum* transcription factor PfNF-YB shows interaction with a CCAAT motif

SUPPLEMENTARY MATERIALS

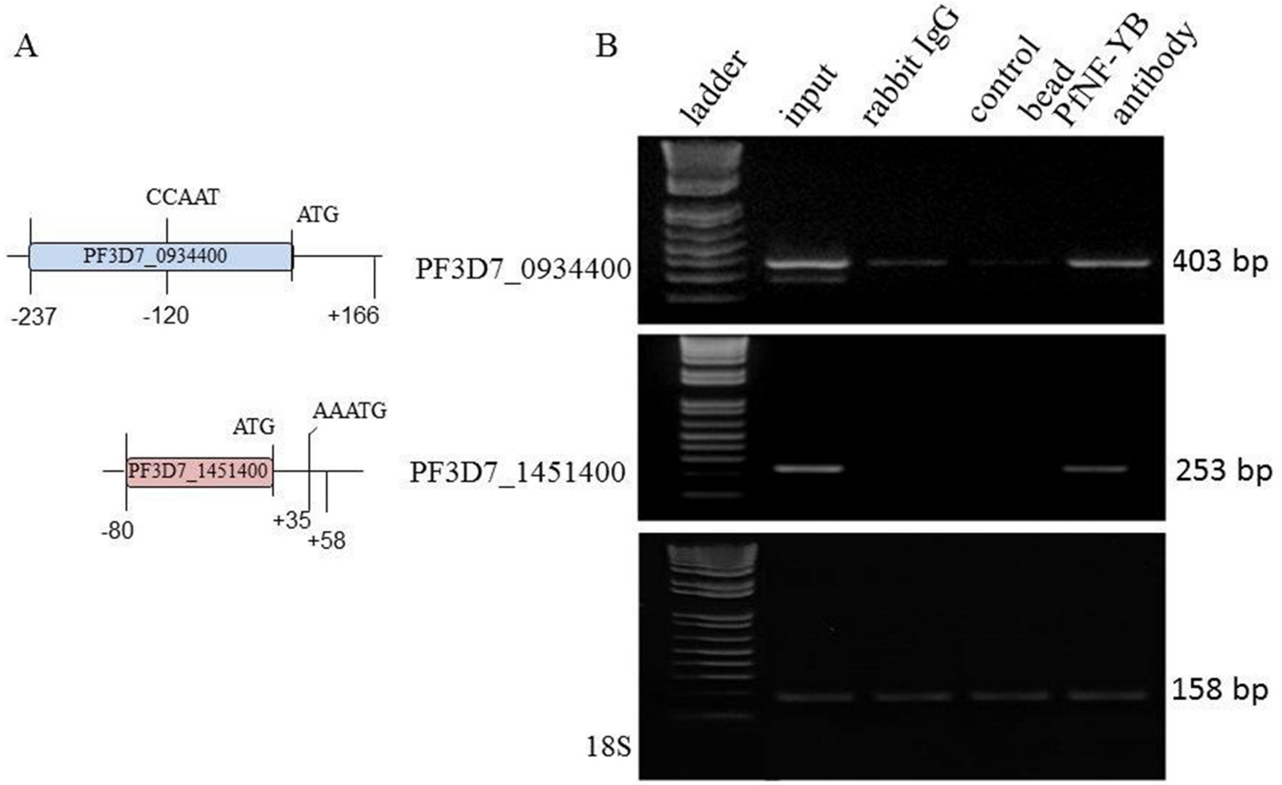

Supplementary Figure 1: PfNF-YB binds to putative promoter region of PFI1665w and PF14_0489 genes. **(A)** Promoter region of PFI1665w and PF14_0489 genes encompassing the potential PfNF-YB binding sites (sequence above). **(B)** Chromatin immunoprecipitation assay using immobilized PfNF-YB antibody, rabbit IgG and control beads. Physical association with PFI1665w (transcription factor with AP2 domain, putative) and PF14_0489 (transcriptional regulatory protein sir2b) genes were tested by PCR and amplification was normalized by reference to 18S rRNA gene. 1Kb DNA ladder was used (Invitrogen).

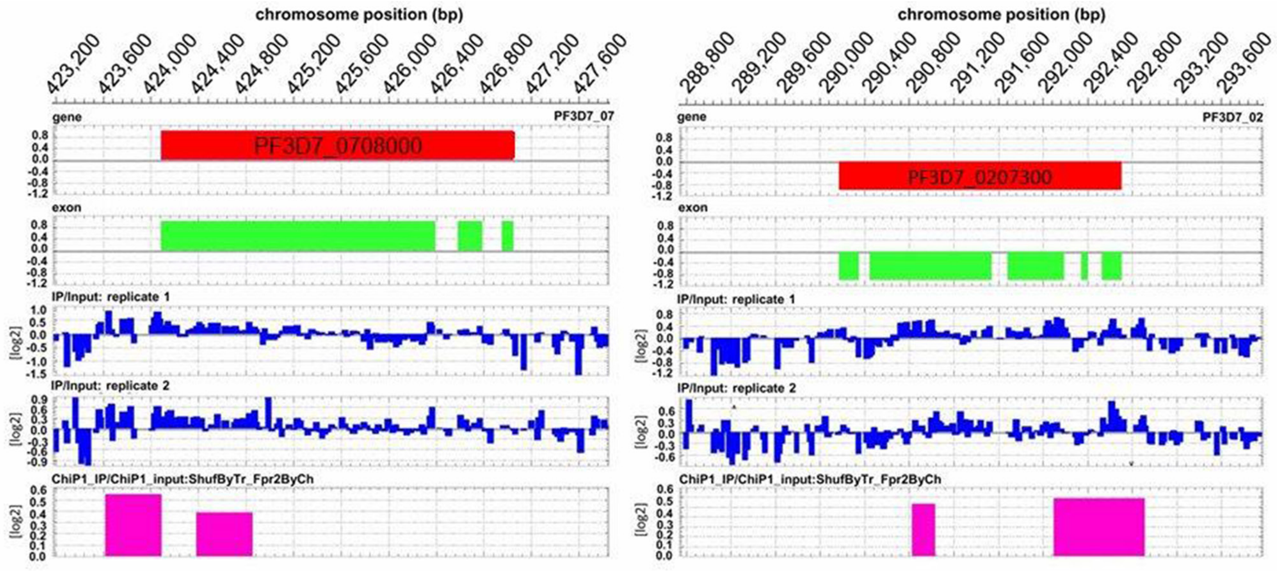

Supplementary Figure 2: PfNF-YB occupancy by high-density ChIP-on-chip assay. The display shows the chromosomal map position and location of DNA enrichment due to PfNF-YB binding for two genes in schizont stage 3D7 parasites. The middle panel with blue scale peaks shows the PfNF-YB occupancy and the lower panel with pink scale peaks illustrates the overlap of candidate promoter regions. PfNF-YB occupancy for each gene was calculated as the average of log_2 ratios of hybridization values for immunoprecipitated and input chromatin. Note that for enriched areas (positive blue peaks) the log2 value was positive, and for non-enriched region the log2 value was next to zero or negative, when using the PfNF-YB custom-made antibody.

Supplementary Table 1: List of PfNF-YB target genes.

See Supplementary Table 1

Supplementary Table 2: Biological functions for PfNF-YB target genes.

See Supplementary Table 2

Supplementary Table 3: Primer used in ChIP-chip assay validation

ID	Primers sequence
PF3D7_0312400	5'GGCTACCTAAATATATAGATAAGC3'<br>5'CCATCAAAAATTCCTCATACACA3'
	5'TGTGCAGTCAAATGTGTAAGACC3'
PF3D7_1426900	5'CAAATCCTTCGCTACACAATGA3'
PF3D7_0619400	5'AAACTTGAGAGTTTGTTTAGGAGGT3'<br>5'GGAATTTCTGGGCATGACTT3'
PF3D7_1241600	5'CAGGAGCAATTTCTGGTGCT3'<br>5'CTCGGATCATAAATGGAGAAGG3'
PF3D7_0806800	5'TGGACACACTGGAATGTTTCTT3'<br>5'GTTGAAAGGCTTGAAGGGAAT3'
PF3D7_1312400	5'AGTGAAGAAACGTGGGCAAA3'<br>5'CTCCGAGAAGATTTCGTTTGA3'
PF3D7_1130200	5'CCAGGACCAACAGGTATGGA3'<br>5'TGTTGCTGAAGAAGCTGTTACC3'
PF3D7_0606500	5'GCATGCAGACCAACAAAATG3'<br>5'CTTTTCCCTTCCTTGACAGG3'
PF3D7_1206600	5'TCAACAACTGCTGAATCGAA3'<br>5'GATCGCTTTCATATCCCATAGC3'
PF3D7_1451400	5'GGGCCACTAGGTGAAGAAGA3'<br>5'GTTGATATGCCAGCACCTGA3'
PF3D7_0934400	5'AGCGAAAACAGGTGAATTGC3'<br>5'GGAAGCAAATTCCATGGGTA3'
PF3D7_1020800	5'GGGTTAAGCGTTTAGAAGATGG3'<br>5'TTTGTTCACTTTTGTCTTTATCAGGA3'
PF3D7_1449300	5'CGAATGAAGGACAGGGAGAA3'<br>5'TTGCTCCATTATTATTTTCAACAA3'
PF3D7_0104000	5'TGGTCTGAATGGAGTGCTTG3'<br>5'TCAGCTTCTTCAAAGGTTCTAGG3'
PF3D7_0409600	5'GATCATCCAAGCGGGAACTA3'<br>5'TGTTTGATGGGTTCACATGTCT3'
PF3D7_0509600	5'GCTGTTATTCATTGGGGAAAA3'<br>5'TTCTCATCAACAATATGGTACAAAAA3'
PF3D7_0605600	CACACATTTTATGCGGACCA<br>TGGCTAACTACAGCGTTCTTCA
PF3D7_0416700	5'TGAGGAGAACGTACTGTCTTGC3'<br>5'CCACCCTTAAAAAGTTCGAAAA 3'
PF3D7_0708000	5'TAAGGTCACTTCCTGCTCGT 3'<br>5'AAAATATTTCATATATGCCGCAAAA 3'
PF3D7_1018500	5' TTTATTTCATTTCATTTTTCCTTCC 3'<br>5'TCTGTGTAGCCATTGGGAGA 3'
PF3D7_1224900	5'TGAGAAACATGTTCACATGGATAA3'<br>5'TGCCTTACTGTTCCATATTTCC3'
MAL-18S	5' AACACAAGGAAGTTTAAGGCAACAA 3'<br>5' GCGTGCAGCCTAGTTCA 3'
PF3D7_0632800	5'GGTTACCCACAACGGCATT 3'<br>5'GGCACCACTACCACCACTTT 3'
PF3D7_1372600	5'TTTGACAGTTGATTGGGAAATG 3'<br>5'CGCCCAAAGTATCCAGTAGG 3'
PF3D7_0415300	5'GGAGGGAAAGCAACAACTATTTT 3'<br>5'TGCTCCTTCTTCCGTATGGT 3'

Supplementary Table 4: Common targets of PfNF-YB.

See Supplementary Table 3
