# Supplementary material for: Genome-wide analysis of the human malaria parasite Plasmodium falciparum transcription factor PfNF-YB shows interaction with a CCAAT motif
Source: Oncotarget. 2017 Dec 9;8(69):113987–4001. doi: 10.18632/oncotarget.23053 (PMC5768380; doi:10.18632/oncotarget.23053)
Supplement: Supplementary file 2 [file oncotarget-08-113987-s002.doc]

Supplementary Table 1 List of PfNF-YB target genes

| Start | End | Strand | FC | FDR | Type | Gene ID |  | Description |
| --- | --- | --- | --- | --- | --- | --- | --- | --- |
| 357910 | 359351 | - | 1.94 | 0.015 | unknown function | PF3D7_1207900 |  | conserved Plasmodium protein, unknown function |
| 410521 | 412261 | - | 1.86 | 0.010 | putative | PF3D7_0408300 |  | zinc finger, RAN binding protein, putative |
| 1436778 | 1439337 | - | 1.81 | 0.000 | unknown function | PF3D7_1234400 |  | conserved Plasmodium protein, unknown function |
| 2114614 | 2122473 | - | 1.78 | 0.006 | unknown function | PF3D7_1252100 |  | conserved Plasmodium protein, unknown function |
| 292018 | 292299 | + | 1.77 | 0.011 | unknown function | PF3D7_0805200 |  | conserved Plasmodium protein, unknown function |
| 1361924 | 1363837 | + | 1.76 | 0.009 | coding gene | PF3D7_0934800 |  | cAMP-dependent protein kinase catalytic subunit |
| 1180660 | 1185473 | - | 1.75 | 0.011 | unknown function | PF3D7_1228900 |  | conserved Plasmodium protein, unknown function |
| 366600 | 367464 | + | 1.74 | 0.000 | putative | PF3D7_0308500 |  | activator of Hsp90 ATPase, putative |
| 284577 | 286302 | + | 1.74 | 0.018 | unknown function | PF3D7_1007000 |  | conserved Plasmodium membrane protein, unknown function |
| 1152377 | 1153225 | + | 1.74 | 0.009 | unknown function | PF3D7_1129800 |  | conserved Plasmodium protein, unknown function |
| 299500 | 299757 | + | 1.74 | 0.000 | unknown function | PF3D7_0306600 |  | conserved Plasmodium protein, unknown function |
| 1068512 | 1069281 | + | 1.74 | 0.011 | unknown function | PF3D7_0525700 |  | conserved Plasmodium protein, unknown function |
| 1973529 | 1974233 | + | 1.73 | 0.106 | unknown function | PF3D7_1248000 |  | conserved Plasmodium protein, unknown function |
| 403342 | 405642 | + | 1.71 | 0.015 | putative | PF3D7_0509600 |  | asparagine-tRNA ligase, putative |
| 768789 | 769453 | + | 1.71 | 0.008 | putative | PF3D7_0618300 |  | 60S ribosomal protein L27a, putative |
| 175844 | 176611 | + | 1.70 | 0.039 | coding gene | PF3D7_0104000 |  | thrombospondin-related sporozoite protein |
| 2690255 | 2691193 | - | 1.70 | 0.016 | putative | PF3D7_1367600 |  | ribosome biogenesis protein MRT4, putative |
| 2684077 | 2685470 | + | 1.70 | 0.058 | putative | PF3D7_1465900 |  | 40S ribosomal protein S3, putative |
| 2483087 | 2483686 | + | 1.70 | 0.076 | unknown function | PF3D7_1362000 |  | conserved Plasmodium protein, unknown function |
| 1668534 | 1669906 | + | 1.70 | 0.009 | unknown function | PF3D7_1440900 |  | conserved Plasmodium protein, unknown function |
| 403717 | 405263 | + | 1.69 | 0.009 | putative | PF3D7_0807900 |  | tyrosyl-tRNA synthetase, putative |
| 552298 | 553435 | - | 1.68 | 0.016 | unknown function | PF3D7_0114200 |  | Plasmodium exported protein (hyp4), unknown function |
| 162065 | 162900 | - | 1.68 | 0.025 | unknown function | PF3D7_0203400 |  | conserved Plasmodium protein, unknown function |
| 78187 | 78781 | + | 1.68 | 0.009 | unknown function | PF3D7_0601900 |  | conserved Plasmodium protein, unknown function |
| 1015869 | 1018445 | + | 1.68 | 0.042 | unknown function | PF3D7_0624900 |  | conserved Plasmodium protein, unknown function |
| 953355 | 955990 | + | 1.67 | 0.015 | putative | PF3D7_1322500 |  | DHHC-type zinc finger protein, putative |
| 2655426 | 2657900 | - | 1.67 | 0.009 | unknown function | PF3D7_1465600 |  | conserved Plasmodium protein, unknown function |
| 382685 | 383968 | + | 1.66 | 0.031 | coding gene | PF3D7_0807300 |  | Rab GTPase 18 |
| 278720 | 283289 | - | 1.66 | 0.009 | coding gene | PF3D7_0405100 |  | Sec24 subunit b |
| 795525 | 797471 | + | 1.66 | 0.000 | putative | PF3D7_0519300 |  | cytochrome c oxidase assembly protein (heme A: farnesyltransferase), putative |
| 798034 | 798773 | - | 1.66 | 0.000 | putative | PF3D7_0519400 |  | 40S ribosomal protein S24, putative |
| 275741 | 279333 | - | 1.66 | 0.009 | putative | PF3D7_1206200 |  | eukaryotic translation initiation factor 3 subunit 8, putative |
| 1067226 | 1068544 | + | 1.66 | 0.009 | unknown function | PF3D7_0824500 |  | conserved Plasmodium protein, unknown function |
| 735920 | 736812 | - | 1.65 | 0.016 | putative | [PF3D7_1018500](http://plasmodb.org/plasmo/showRecord.do?name=GeneRecordClasses.GeneRecordClass&source_id=PF3D7_1018500&project_id=PlasmoDB) |  | PHF5-like protein, putative |
| 1349779 | 1350381 | + | 1.65 | 0.105 | putative | PF3D7_0934400 |  | transcription factor with AP2 domain(s), putative |
| 2714187 | 2715711 | + | 1.65 | 0.076 | unknown function | PF3D7_1368300 |  | conserved Plasmodium protein, unknown function |
| 467721 | 468539 | - | 1.64 | 0.018 | coding gene | PF3D7_0709200 |  | Cg6 protein |
| 1636587 | 1637864 | + | 1.64 | 0.016 | putative | PF3D7_1341300 |  | 60S ribosomal protein L18-2, putative |
| 527879 | 528919 | - | 1.64 | 0.029 | unknown function | PF3D7_0710300 |  | conserved Plasmodium membrane protein, unknown function |
| 281907 | 287345 | + | 1.64 | 0.192 | unknown function | PF3D7_0207100 |  | conserved Plasmodium protein, unknown function |
| 691559 | 693560 | - | 1.63 | 0.168 | coding gene | PF3D7_0916500 |  | apicoplast Ufd1 precursor |
| 1021848 | 1023610 | - | 1.63 | 0.045 | coding gene | PF3D7_0925400 |  | protein phosphatase-beta |
| 1010824 | 1011334 | - | 1.63 | 0.105 | putative | PF3D7_1224900 |  | splicing factor 3b subunit, putative |
| 1893058 | 1893892 | + | 1.63 | 0.016 | putative | PF3D7_1245300 |  | ubiquitin conjugating enzyme E2, putative |
| 2108810 | 2112724 | + | 1.62 | 0.010 | coding gene | PF3D7_1451400 |  | transcriptional regulatory protein sir2b |
| 343913 | 349351 | - | 1.61 | 0.096 | coding gene | PF3D7_1107900 |  | mechanosensitive ion channel protein |
| 272317 | 274739 | - | 1.61 | 0.016 | putative | PF3D7_1206100 |  | IMP-specific 5'-nucleotidase, putative, haloacid dehalogenase hydrolase, putative |
| 1209945 | 1211973 | - | 1.61 | 0.010 | unknown function | PF3D7_1328600 |  | conserved Plasmodium protein, unknown function |
| 2502481 | 2503547 | + | 1.61 | 0.016 | unknown function | PF3D7_1362600 |  | conserved Plasmodium protein, unknown function |
| 729986 | 730506 | - | 1.61 | 0.016 | unknown function | PF3D7_0715100 |  | conserved Plasmodium protein, unknown function |
| 230528 | 234641 | - | 1.61 | 0.018 | unknown function | PF3D7_0105600 |  | conserved Plasmodium protein, unknown function |
| 455997 | 456488 | + | 1.61 | 0.045 | unknown function | PF3D7_0112100 |  | conserved Plasmodium protein, unknown function |
| 148237 | 155476 | - | 1.61 | 0.016 | unknown function | PF3D7_1202600 |  | conserved protein, unknown function |
| 3207562 | 3207834 | + | 1.60 | 0.031 | coding gene | PF3D7_1477800 |  | acyl-CoA binding protein |
| 1301219 | 1301764 | + | 1.60 | 0.016 | coding gene | PF3D7_0532100 |  | early transcribed membrane protein 5 |
| 475579 | 476058 | + | 1.60 | 0.145 | ncRNA | PF3D7_0809300 |  | PF08TR001 |
| 1003087 | 1004625 | + | 1.60 | 0.016 | putative | PF3D7_0422000 |  | steroid dehydrogenase, putative |
| 937226 | 938032 | + | 1.60 | 0.016 | putative | PF3D7_0522500 |  | mitochondrial ribosomal protein L17 precursor, putative |
| 1019580 | 1019897 | - | 1.60 | 0.129 | putative | PF3D7_0524700 |  | mitochondrial import receptor subunit tom22, putative |
| 228129 | 234832 | - | 1.60 | 0.010 | putative | PF3D7_0605600 |  | nucleoside diphosphate kinase, putative |
| 447919 | 452040 | + | 1.60 | 0.029 | putative | PF3D7_0909900 |  | helicase with Zn-finger motif, putative |
| 262672 | 263925 | - | 1.60 | 0.075 | unknown function | PF3D7_0506400 |  | conserved Plasmodium protein, unknown function |
| 636093 | 639510 | - | 1.59 | 0.023 | coding gene | PF3D7_1116800 |  | heat shock protein 101 |
| 3220861 | 3221511 | + | 1.59 | 0.168 | coding gene | PF3D7_1478300 |  | hypothetical protein |
| 1101735 | 1102853 | - | 1.59 | 0.000 | coding gene | PF3D7_0627500 |  | 4-methyl-5(B-hydroxyethyl)-thiazol monophosphate biosynthesis enzyme |
| 822601 | 824592 | - | 1.59 | 0.025 | putative | PF3D7_1020400 |  | methyltransferase, putative |
| 1021290 | 1021772 | - | 1.59 | 0.025 | putative | PF3D7_1024200 |  | RNA binding protein, putative |
| 1126984 | 1128973 | + | 1.59 | 0.025 | putative | PF3D7_1428600 |  | peptide chain release factor 1, putative |
| 92503 | 96665 | + | 1.59 | 0.036 | putative | PF3D7_0602100 |  | ATP-dependent RNA helicase, putative |
| 1023203 | 1024867 | - | 1.59 | 0.045 | unknown function | PF3D7_1024400 |  | conserved Plasmodium protein, unknown function |
| 1241765 | 1242975 | - | 1.59 | 0.014 | unknown function | PF3D7_1030400 |  | conserved Plasmodium protein, unknown function |
| 535590 | 537740 | - | 1.58 | 0.048 | putative | PF3D7_0810500 |  | protein phosphatase, putative |
| 942645 | 943868 | + | 1.58 | 0.023 | putative | PF3D7_0522700 |  | iron-sulfur assembly protein, putative |
| 944179 | 944926 | - | 1.58 | 0.023 | putative | PF3D7_0522800 |  | G10 protein, putative |
| 294994 | 299346 | - | 1.58 | 0.168 | putative | PF3D7_1206600 |  | DNA-directed RNA polymerase III subunit, putative |
| 2064972 | 2066025 | + | 1.58 | 0.006 | putative | PF3D7_1250300 |  | Hbeta58/Vps26 protein homolog, putative |
| 2461824 | 2464315 | - | 1.58 | 0.023 | unknown function | [PF3D7_1361500.1](http://plasmodb.org/plasmo/showRecord.do?name=GeneRecordClasses.GeneRecordClass&source_id=PF3D7_1361500.1&project_id=PlasmoDB) |  | conserved Plasmodium protein, unknown function |
| 1185667 | 1186323 | + | 1.58 | 0.017 | unknown function | PF3D7_0827400 |  | conserved Plasmodium protein, unknown function |
| 1979773 | 1980186 | - | 1.58 | 0.025 | unknown function | PF3D7_1248300 |  | conserved Plasmodium membrane protein, unknown function |
| 270738 | 274787 | - | 1.57 | 0.018 | coding gene | PF3D7_0905400 |  | high molecular weight rhoptry protein 3 |
| 795529 | 797460 | + | 1.57 | 0.031 | coding gene | PF3D7_0919400 |  | protein disulfide isomerase |
| 1231320 | 1237076 | + | 1.57 | 0.006 | putative | PF3D7_0727800 |  | cation transporting ATPase, putative |
| 383044 | 384793 | - | 1.57 | 0.197 | putative | PF3D7_1009500 |  | metallopeptidase, putative |
| 734693 | 734962 | - | 1.57 | 0.006 | putative | PF3D7_0416700 |  | PfMNL-2 CISD1-like iron-sulfur protein, putative |
| 199395 | 201329 | + | 1.57 | 0.042 | unknown function | PF3D7_0703600 |  | conserved Plasmodium protein, unknown function |
| 458600 | 461695 | + | 1.56 | 0.062 | coding gene | PF3D7_0709000 |  | chloroquine resistance transporter |
| 493278 | 493472 | + | 1.56 | 0.026 | unknown function | PF3D7_0709800 |  | conserved Plasmodium protein, unknown function |
| 596288 | 597202 | - | 1.56 | 0.031 | unknown function | PF3D7_1115800 |  | conserved Plasmodium protein, unknown function |
| 756191 | 760434 | + | 1.56 | 0.026 | unknown function | PF3D7_1120000 |  | conserved Plasmodium protein, unknown function |
| 1065783 | 1066567 | + | 1.56 | 0.018 | unknown function | PF3D7_1127400 |  | conserved Plasmodium protein, unknown function |
| 1878601 | 1882520 | - | 1.56 | 0.031 | unknown function | PF3D7_1445800 |  | conserved Plasmodium membrane protein, unknown function |
| 2406604 | 2410095 | + | 1.56 | 0.009 | unknown function | PF3D7_1458600 |  | conserved Plasmodium protein, unknown function |
| 840211 | 843402 | + | 1.55 | 0.026 | coding gene | PF3D7_1020800 |  | dihydrolipoamide acyltransferase component E2 |
| 831139 | 832587 | + | 1.55 | 0.031 | putative | PF3D7_0818200 |  | 14-3-3 protein, putative |
| 364381 | 367542 | - | 1.55 | 0.031 | putative | PF3D7_0806800 |  | vacuolar proton translocating ATPase subunit A, putative |
| 662008 | 664297 | - | 1.55 | 0.039 | putative | PF3D7_0414500 |  | RNA binding protein, putative |
| 864602 | 869234 | - | 1.55 | 0.031 | unknown function | PF3D7_0819000 |  | conserved Plasmodium protein, unknown function |
| 540677 | 546046 | - | 1.55 | 0.162 | unknown function | PF3D7_1413700 |  | conserved Plasmodium protein, unknown function |
| 2892159 | 2895937 | - | 1.55 | 0.106 | unknown function | PF3D7_1470800 |  | conserved Plasmodium protein, unknown function |
| 791175 | 793411 | + | 1.55 | 0.031 | unknown function | PF3D7_0417700 |  | conserved Plasmodium protein, unknown function |
| 158230 | 160008 | + | 1.55 | 0.058 | unknown function | PF3D7_1202700 |  | conserved Plasmodium protein, unknown function |
| 2285049 | 2290163 | + | 1.54 | 0.042 | coding gene | PF3D7_1455800 |  | LCCL domain-containing protein |
| 837111 | 837981 | - | 1.54 | 0.042 | putative | PF3D7_0818400 |  | nucleolar preribosomal assembly protein, putative |
| 1145821 | 1146793 | - | 1.54 | 0.042 | putative | PF3D7_1027400 |  | DNA-directed RNA polymerase II, putative |
| 1135535 | 1136381 | + | 1.54 | 0.037 | putative | [PF3D7_0928000.1](http://plasmodb.org/plasmo/showRecord.do?name=GeneRecordClasses.GeneRecordClass&source_id=PF3D7_0928000.1&project_id=PlasmoDB) |  | cytochrome c oxidase, putative |
| 356083 | 360434 | + | 1.54 | 0.029 | unknown function | PF3D7_1108100 |  | conserved Plasmodium protein, unknown function |
| 220404 | 221810 | - | 1.54 | 0.035 | unknown function | PF3D7_0605200 |  | conserved Plasmodium protein, unknown function |
| 349942 | 350511 | - | 1.54 | 0.031 | unknown function | PF3D7_0608400 |  | conserved Plasmodium protein, unknown function |
| 803371 | 814549 | - | 1.54 | 0.030 | unknown function | PF3D7_0619300 |  | conserved Plasmodium protein, unknown function |
| 1458380 | 1459850 | + | 1.54 | 0.105 | unknown function | PF3D7_0936800 |  | Plasmodium exported protein (PHISTc), unknown function |
| 1669725 | 1670987 | - | 1.53 | 0.065 | coding gene | PF3D7_1342500 |  | sporozoite protein essential for cell traversal |
| 288872 | 294766 | - | 1.53 | 0.042 | coding gene | PF3D7_0405300 |  | sequestrin |
| 1065787 | 1066674 | + | 1.53 | 0.032 | coding gene | PF3D7_1226300 |  | cof-like hydrolase, had-superfamily, subfamily iib |
| 1305011 | 1305104 | + | 1.53 | 0.037 | ncRNA | RNAzID:1692 |  | RNAzID:1692 |
| 1300104 | 1305492 | - | 1.53 | 0.037 | putative | PF3D7_1331100 |  | DEAD box helicase, putative |
| 1601893 | 1603749 | - | 1.53 | 0.037 | putative | PF3D7_1340000 |  | secreted ookinete protein, putative |
| 947012 | 954427 | + | 1.53 | 0.026 | putative | PF3D7_0720700 |  | phosphoinositide-binding protein, putative |
| 1936831 | 1937317 | + | 1.53 | 0.071 | putative | PF3D7_1447300 |  | mitochondrial ribosomal protein S14 precursor, putative |
| 376855 | 378452 | - | 1.53 | 0.096 | putative | PF3D7_0509000 |  | SNAP protein, putative |
| 1332497 | 1335790 | + | 1.53 | 0.145 | unknown function | PF3D7_1332200 |  | conserved Plasmodium protein, unknown function |
| 101005 | 101961 | + | 1.53 | 0.029 | unknown function | PF3D7_0301800 |  | Plasmodium exported protein, unknown function |
| 708925 | 710754 | - | 1.53 | 0.042 | unknown function | PF3D7_0317400 |  | conserved Plasmodium protein, unknown function |
| 1245525 | 1249160 | + | 1.53 | 0.032 | unknown function | PF3D7_1230200 |  | conserved Plasmodium protein, unknown function |
| 1217343 | 1221788 | + | 1.52 | 0.168 | coding gene | PF3D7_0828200 |  | leucine-tRNA ligase |
| 285541 | 288452 | + | 1.52 | 0.042 | coding gene | PF3D7_0405200 |  | ag-1 blood stage membrane protein homologue |
| 140710 | 141471 | + | 1.52 | 0.042 | coding gene | PF3D7_0503400 |  | actin-depolymerizing factor |
| 489904 | 490818 | - | 1.52 | 0.168 | putative | PF3D7_0212200 |  | mitochondrial ribosomal protein L12 precursor, putative |
| 747565 | 749364 | - | 1.52 | 0.129 | putative | [PF3D7_0218300](http://plasmodb.org/plasmo/showRecord.do?name=GeneRecordClasses.GeneRecordClass&source_id=PF3D7_0218300&project_id=PlasmoDB) |  | apicoplast RNA methyltransferase precursor, putative |
| 838425 | 839080 | + | 1.52 | 0.042 | putative | PF3D7_0520300 |  | U6 snRNA-associated sm-like protein lsm2, putative |
| 1178269 | 1178883 | - | 1.52 | 0.045 | putative | PF3D7_0528700 |  | cyclophilin, putative |
| 523652 | 525100 | - | 1.52 | 0.042 | putative | PF3D7_0612600 |  | PP-loop family protein, putative |
| 815619 | 818244 | - | 1.52 | 0.143 | putative | PF3D7_0619400 |  | cell division cycle protein 48 homologue, putative |
| 1089901 | 1092420 | + | 1.52 | 0.168 | putative | PF3D7_0926700 |  | NAD synthase, putative |
| 571006 | 573305 | - | 1.52 | 0.071 | unknown function | PF3D7_1115100 |  | conserved Plasmodium protein, unknown function |
| 471629 | 474236 | - | 1.52 | 0.105 | unknown function | PF3D7_0410000 |  | conserved Plasmodium protein, unknown function |
| 750659 | 752433 | + | 1.52 | 0.042 | unknown function | PF3D7_0518000 |  | conserved protein, unknown function |
| 402718 | 407238 | + | 1.52 | 0.039 | unknown function | PF3D7_0609500 |  | conserved Plasmodium protein, unknown function |
| 764664 | 765489 | - | 1.52 | 0.081 | unknown function | PF3D7_0618200 |  | conserved Plasmodium protein, unknown function |
| 307300 | 309036 | - | 1.52 | 0.058 | unknown function | PF3D7_0906200 |  | conserved Plasmodium protein, unknown function |
| 691249 | 691369 | + | 1.51 | 0.129 | ncRNA | RNAzID:345 |  | RNAzID:345 |
| 1273802 | 1280849 | + | 1.51 | 0.039 | putative | PF3D7_0728600 |  | zinc finger, C3HC4 type, putative |
| 277621 | 279168 | + | 1.51 | 0.105 | putative | PF3D7_1006800 |  | RNA binding protein, putative |
| 715877 | 717436 | + | 1.51 | 0.053 | putative | PF3D7_1017900 |  | 26s proteasome regulatory subunit p55, putative |
| 320128 | 321205 | - | 1.51 | 0.058 | putative | PF3D7_1107500 |  | prefoldin, putative |
| 2444409 | 2446115 | - | 1.51 | 0.023 | putative | PF3D7_1459700 |  | pyridoxal 5'-phosphate synthase, putative |
| 1028034 | 1029648 | + | 1.51 | 0.042 | unknown function | PF3D7_0723200 |  | conserved Plasmodium protein, unknown function |
| 460334 | 461992 | - | 1.51 | 0.058 | unknown function | PF3D7_1011900 |  | conserved Plasmodium protein, unknown function |
| 983950 | 985618 | - | 1.51 | 0.058 | unknown function | PF3D7_1125000 |  | conserved Plasmodium protein, unknown function |
| 1588895 | 1593074 | + | 1.51 | 0.058 | unknown function | PF3D7_1439200 |  | conserved Plasmodium protein, unknown function |
| 2922288 | 2924494 | - | 1.51 | 0.036 | unknown function | PF3D7_1471500 |  | conserved Plasmodium membrane protein, unknown function |
| 226066 | 226419 | + | 1.50 | 0.192 | coding gene | PF3D7_1105100 |  | histone H2B |
| 520829 | 523114 | - | 1.50 | 0.058 | coding gene | PF3D7_0312400 |  | glycogen synthase kinase 3 |
| 424269 | 427721 | - | 1.50 | 0.053 | coding gene | PF3D7_0408700 |  | perforin like protein 1 |
| 459031 | 462468 | - | 1.50 | 0.168 | coding gene | PF3D7_0409600 |  | replication protein A large subunit |
| 1685083 | 1685400 | - | 1.50 | 0.026 | coding gene | PF3D7_1240100 |  | early transcribed membrane protein 12 |
| 221946 | 223431 | + | 1.50 | 0.106 | putative | PF3D7_1004800 |  | ADP/ATP carrier protein, putative |
| 1682824 | 1684449 | + | 1.50 | 0.026 | putative | PF3D7_1240000 |  | 3-hydroxyisobutyryl-coenzyme A hydrolase, putative |
| 1774889 | 1776570 | + | 1.50 | 0.045 | putative | PF3D7_1241600 |  | mitochondrial carrier protein, putative |
| 1064145 | 1066612 | + | 1.50 | 0.175 | unknown function | PF3D7_1325500 |  | conserved Plasmodium protein, unknown function |
| 2235594 | 2236337 | + | 1.50 | 0.045 | unknown function | PF3D7_1356500 |  | conserved Plasmodium protein, unknown function |
| 325462 | 331944 | - | 1.50 | 0.036 | unknown function | PF3D7_0307700 |  | conserved Plasmodium protein, unknown function |
| 1116974 | 1118115 | - | 1.50 | 0.032 | unknown function | PF3D7_0424600 |  | Plasmodium exported protein (PHISTb), unknown function |
| 1140213 | 1141012 | + | 1.50 | 0.058 | unknown function | PF3D7_0425200 |  | Plasmodium exported protein (hyp15), unknown function |
| 600947 | 601557 | - | 1.50 | 0.058 | unknown function | PF3D7_1214000 |  | conserved Plasmodium protein, unknown function |
| 1003193 | 1007578 | - | 1.50 | 0.058 | unknown function | PF3D7_1224700 |  | conserved Plasmodium protein, unknown function |
| 1458221 | 1459170 | + | 1.50 | 0.145 | unknown function | PF3D7_1234900 |  | conserved Plasmodium protein, unknown function |
| 475326 | 476562 | - | 1.49 | 0.069 | coding gene | PF3D7_1012400 |  | hypoxanthine phosphoribosyltransferase |
| 247637 | 249163 | + | 1.49 | 0.086 | putative | PF3D7_0605900 |  | LC polyunsaturated fatty acid elongation enzyme, putative |
| 1199854 | 1200996 | - | 1.49 | 0.058 | putative | PF3D7_0629200 |  | DnaJ protein, putative |
| 416365 | 417309 | + | 1.49 | 0.096 | unknown function | PF3D7_0509900 |  | conserved Plasmodium protein, unknown function |
| 680222 | 681928 | - | 1.49 | 0.044 | unknown function | PF3D7_0916300 |  | conserved Plasmodium protein, unknown function |
| 1161070 | 1162529 | + | 1.49 | 0.076 | unknown function | PF3D7_0929000 |  | conserved Plasmodium protein, unknown function |
| 615743 | 617185 | - | 1.48 | 0.018 | coding gene | [PF3D7_1116200.1](http://plasmodb.org/plasmo/showRecord.do?name=GeneRecordClasses.GeneRecordClass&source_id=PF3D7_1116200.1&project_id=PlasmoDB) |  | SNO glutamine amidotransferase family protein |
| 1639420 | 1641703 | + | 1.48 | 0.076 | coding gene | PF3D7_1440300 |  | delta-aminolevulinic acid dehydratase |
| 595721 | 596947 | - | 1.48 | 0.058 | pseudogene | PF3D7_0712200 |  | rifin, pseudogene |
| 1243373 | 1246345 | + | 1.48 | 0.076 | putative | PF3D7_1329400 |  | AMP deaminase, putative |
| 1449687 | 1451447 | - | 1.48 | 0.069 | putative | PF3D7_1136800 |  | DnaJ protein, putative |
| 1049992 | 1050258 | - | 1.48 | 0.081 | putative | PF3D7_1426900 |  | ubiquinol-cytochrome c reductase hinge protein, putative |
| 1378291 | 1402608 | - | 1.48 | 0.168 | putative | PF3D7_1434500 |  | dynein-related AAA-type ATPase, putative |
| 1498701 | 1499435 | - | 1.48 | 0.076 | putative | PF3D7_1436800 |  | ATP-dependent Clp protease proteolytic subunit, putative |
| 1639420 | 1647110 | + | 1.48 | 0.076 | putative | PF3D7_1440200 |  | stromal-processing peptidase, putative |
| 2151759 | 2153550 | - | 1.48 | 0.105 | putative | PF3D7_1452500 |  | SNARE protein, putative |
| 939658 | 943720 | - | 1.48 | 0.039 | putative | PF3D7_0623100 |  | coronin binding protein, putative |
| 670440 | 673259 | + | 1.48 | 0.076 | putative | PF3D7_1216900 |  | DNA-binding chaperone, putative |
| 2560960 | 2563443 | + | 1.48 | 0.076 | unknown function | PF3D7_1364000 |  | conserved Plasmodium protein, unknown function |
| 749899 | 750258 | + | 1.48 | 0.045 | unknown function | PF3D7_0715700 |  | conserved Plasmodium protein, unknown function |
| 429690 | 430861 | - | 1.48 | 0.023 | unknown function | PF3D7_1410700 |  | conserved Plasmodium protein, unknown function |
| 1830952 | 1832746 | + | 1.48 | 0.076 | unknown function | PF3D7_1444400 |  | conserved Plasmodium protein, unknown function |
| 2577909 | 2579314 | + | 1.48 | 0.076 | unknown function | PF3D7_1463500 |  | conserved Plasmodium protein, unknown function |
| 2766044 | 2766552 | + | 1.48 | 0.095 | unknown function | PF3D7_1467700 |  | conserved Plasmodium protein, unknown function |
| 3023787 | 3038767 | - | 1.48 | 0.045 | unknown function | PF3D7_1474200 |  | conserved Plasmodium membrane protein, unknown function |
| 3120789 | 3121784 | + | 1.48 | 0.076 | unknown function | PF3D7_1475800 |  | conserved Plasmodium protein, unknown function |
| 681054 | 682022 | - | 1.48 | 0.081 | unknown function | PF3D7_1416700 |  | conserved Plasmodium protein, unknown function |
| 506065 | 510777 | + | 1.48 | 0.175 | unknown function | PF3D7_0411000 |  | conserved Plasmodium protein, unknown function |
| 517277 | 519445 | + | 1.48 | 0.039 | unknown function | PF3D7_0411300 |  | conserved Plasmodium protein, unknown function |
| 521223 | 521933 | + | 1.48 | 0.096 | unknown function | PF3D7_0612400 |  | conserved Plasmodium protein, unknown function |
| 1787224 | 1788819 | - | 1.48 | 0.129 | unknown function | PF3D7_1242000 |  | conserved Plasmodium protein, unknown function |
| 2409657 | 2421594 | + | 1.47 | 0.045 | coding gene | PF3D7_1360500 |  | guanylyl cyclase beta |
| 667815 | 667878 | + | 1.47 | 0.129 | ncRNA | RNAzID:2906 |  | RNAzID:2906 |
| 665976 | 667547 | - | 1.47 | 0.129 | putative | PF3D7_0316600 |  | formate-nitrite transporter, putative |
| 812239 | 813525 | + | 1.47 | 0.069 | putative | PF3D7_0319500 |  | RNA binding protein, putative |
| 1246283 | 1252150 | + | 1.47 | 0.071 | unknown function | PF3D7_0829000 |  | conserved Plasmodium membrane protein, unknown function |
| 1318526 | 1319224 | - | 1.47 | 0.076 | unknown function | PF3D7_0532600 |  | Plasmodium exported protein, unknown function |
| 657935 | 661785 | - | 1.47 | 0.076 | unknown function | PF3D7_0615800 |  | conserved Plasmodium protein, unknown function |
| 1165219 | 1166169 | + | 1.46 | 0.109 | coding gene | PF3D7_1130200 |  | 60S ribosomal protein P0 |
| 1548861 | 1556664 | + | 1.46 | 0.145 | coding gene | PF3D7_1438400 |  | metacaspase-like protein |
| 1954544 | 1957618 | - | 1.46 | 0.096 | coding gene | PF3D7_1447900 |  | multidrug resistance protein 2 (heavy metal transport family) |
| 777548 | 780739 | + | 1.46 | 0.145 | coding gene | PF3D7_0918900 |  | gamma-glutamylcysteine synthetase |
| 424085 | 427050 | + | 1.46 | 0.045 | putative | PF3D7_0708000 |  | cytoskeleton associated protein, putative |
| 374864 | 377611 | + | 1.46 | 0.096 | putative | PF3D7_1009200 |  | small subunit rRNA synthesis-associated protein, putative |
| 1053640 | 1054331 | - | 1.46 | 0.096 | putative | PF3D7_1126900 |  | small nuclear ribonucleoprotein F, putative |
| 1997485 | 1998246 | - | 1.46 | 0.096 | putative | PF3D7_1448600 |  | SNARE protein, putative |
| 2016901 | 2019503 | + | 1.46 | 0.081 | putative | PF3D7_1449300 |  | transcription factor IIIb, putative |
| 2886824 | 2889143 | - | 1.46 | 0.106 | putative | PF3D7_1470600 |  | RAP protein, putative |
| 2902072 | 2903475 | + | 1.46 | 0.139 | putative | PF3D7_1471000 |  | RNA 3'-terminal phosphate cyclase-like protein, putative |
| 265835 | 268395 | - | 1.46 | 0.045 | putative | PF3D7_0606500 |  | polypyrimidine tract binding protein, putative |
| 966785 | 968059 | - | 1.46 | 0.096 | putative | PF3D7_0923900 |  | RNA binding protein, putative |
| 971810 | 972544 | + | 1.46 | 0.076 | unknown function | PF3D7_0721100 |  | conserved Plasmodium protein, unknown function |
| 2519140 | 2523144 | - | 1.46 | 0.096 | unknown function | PF3D7_1462100 |  | conserved Plasmodium protein, unknown function |
| 833744 | 839302 | + | 1.46 | 0.096 | unknown function | PF3D7_0920400 |  | conserved Plasmodium protein, unknown function |
| 655409 | 657461 | - | 1.45 | 0.062 | coding gene | PF3D7_1315600 |  | phosphatidylinositol synthase |
| 335693 | 336166 | + | 1.45 | 0.096 | coding gene | PF3D7_0406200 |  | sexual stage-specific protein precursor |
| 663054 | 666275 | + | 1.45 | 0.035 | coding gene | PF3D7_1216700 |  | perforin like protein 2 |
| 168969 | 170182 | - | 1.45 | 0.168 | pseudogene | PF3D7_0402700 |  | rifin, pseudogene |
| 1316694 | 1318004 | - | 1.45 | 0.129 | putative | PF3D7_1331600 |  | protein tyrosine phosphatase, putative |
| 216854 | 220231 | - | 1.45 | 0.139 | putative | PF3D7_1304000 |  | chromosome condensation protein, putative |
| 2484190 | 2485808 | - | 1.45 | 0.066 | putative | PF3D7_1362100 |  | 14-3-3 protein, putative |
| 521252 | 524638 | + | 1.45 | 0.053 | putative | PF3D7_1312400 |  | translation initiation factor IF-2, putative |
| 611534 | 619090 | + | 1.45 | 0.062 | putative | PF3D7_1314200 |  | telomerase reverse transcriptase, putative |
| 2688410 | 2689616 | + | 1.45 | 0.081 | putative | PF3D7_1367500 |  | NADH-cytochrome b5 reductase, putative |
| 667607 | 669502 | + | 1.45 | 0.048 | putative | PF3D7_0414700 |  | GTP binding protein, putative |
| 571056 | 572659 | - | 1.45 | 0.145 | putative | PF3D7_0513400 |  | GTP binding protein, putative |
| 648431 | 650545 | + | 1.45 | 0.112 | putative | PF3D7_1216200 |  | glycerol-3-phosphate dehydrogenase, putative |
| 998345 | 999267 | + | 1.45 | 0.058 | putative | PF3D7_1224500 |  | chromatin assembly protein (ASF1), putative |
| 1820869 | 1822583 | - | 1.45 | 0.106 | putative | PF3D7_1243500 |  | endosome sorting protein (SNF7 homologue), putative |
| 1155223 | 1158106 | + | 1.45 | 0.106 | unknown function | PF3D7_1327500 |  | conserved Plasmodium protein, unknown function |
| 463587 | 464204 | - | 1.45 | 0.062 | unknown function | PF3D7_1310500 |  | conserved Plasmodium protein, unknown function |
| 775232 | 783638 | - | 1.45 | 0.048 | unknown function | PF3D7_1120600 |  | conserved Plasmodium protein, unknown function |
| 1371869 | 1373254 | + | 1.45 | 0.112 | unknown function | PF3D7_1333500 |  | conserved Plasmodium protein, unknown function |
| 977376 | 980636 | - | 1.45 | 0.096 | unknown function | PF3D7_0323400 |  | conserved Plasmodium protein, unknown function |
| 55841 | 56872 | + | 1.45 | 0.075 | unknown function | PF3D7_0501000 |  | Plasmodium exported protein, unknown function |
| 1933748 | 1935454 | - | 1.45 | 0.095 | unknown function | PF3D7_1246500 |  | conserved Plasmodium protein, unknown function |
| 3290888 | 3291436 | + | 1.44 | 0.081 | pseudogene | PF3D7_1480100 |  | erythrocyte membrane protein 1 (PfEMP1), pseudogene |
| 1310747 | 1313029 | + | 1.44 | 0.095 | putative | PF3D7_0729500 |  | mRNA (N6-adenosine)-methyltransferase, putative |
| 463492 | 467005 | + | 1.44 | 0.044 | putative | PF3D7_1012000 |  | ubiquitin-protein ligase e3, putative |
| 1557470 | 1560100 | - | 1.44 | 0.139 | putative | PF3D7_1438500 |  | cleavage and polyadenylation specifity factor, putative |
| 1780935 | 1783523 | - | 1.44 | 0.139 | putative | PF3D7_1443500 |  | CAF1 family ribonuclease, putative |
| 576215 | 577141 | - | 1.44 | 0.175 | putative | PF3D7_0314400 |  | serine/threonine protein phosphatase, putative |
| 237808 | 244518 | - | 1.44 | 0.105 | putative | PF3D7_0605800 |  | DNA repair-like protein, putative |
| 392987 | 394607 | + | 1.44 | 0.096 | putative | PF3D7_0609200 |  | citrate synthase-like protein, putative |
| 1196042 | 1198039 | - | 1.44 | 0.048 | putative | PF3D7_0629100 |  | nicotinate phosphoribosyltransferase, putative |
| 1207531 | 1210731 | - | 1.44 | 0.109 | unknown function | PF3D7_0727100 |  | conserved Plasmodium protein, unknown function |
| 596496 | 600412 | - | 1.44 | 0.162 | unknown function | PF3D7_1014800 |  | conserved Plasmodium protein, unknown function |
| 766167 | 771953 | - | 1.44 | 0.086 | unknown function | PF3D7_1019100 |  | conserved Plasmodium protein, unknown function |
| 1067650 | 1086990 | - | 1.44 | 0.145 | unknown function | PF3D7_1025500 |  | conserved Plasmodium protein, unknown function |
| 152538 | 160452 | - | 1.44 | 0.109 | unknown function | PF3D7_0603800 |  | conserved Plasmodium protein, unknown function |
| 1011430 | 1014570 | - | 1.44 | 0.182 | unknown function | PF3D7_0624800 |  | conserved Plasmodium protein, unknown function |
| 1621487 | 1622613 | - | 1.43 | 0.044 | coding gene | PF3D7_1340700 |  | Rab GTPase 11b |
| 480586 | 482799 | + | 1.43 | 0.105 | coding gene | PF3D7_1210900 |  | GPI mannosyltransferase I |
| 651391 | 651849 | - | 1.43 | 0.139 | coding gene | PF3D7_1216300 |  | signal recognition particle SRP19 |
| 976879 | 977935 | - | 1.43 | 0.139 | putative | PF3D7_1124800 |  | nuclear preribosomal assembly protein, putative |
| 1104214 | 1106503 | - | 1.43 | 0.139 | putative | PF3D7_1128400 |  | farnesyl pyrophosphate synthase, putative |
| 1755435 | 1755932 | + | 1.43 | 0.139 | putative | PF3D7_1144000 |  | 40S ribosomal protein S21e, putative |
| 1218047 | 1218941 | - | 1.43 | 0.197 | putative | PF3D7_1431000 |  | mitochondrial ribosomal protein L17-2 precursor, putative |
| 222693 | 224989 | - | 1.43 | 0.139 | putative | PF3D7_1205100 |  | UGA suppressor tRNA-associated antigenic protein, putative |
| 270297 | 271211 | - | 1.43 | 0.105 | putative | PF3D7_1206000 |  | protein phosphatase, putative |
| 1977475 | 1978925 | + | 1.43 | 0.139 | putative | PF3D7_1248200 |  | RNA binding protein, putative |
| 2581367 | 2591164 | + | 1.43 | 0.139 | unknown function | PF3D7_1364400 |  | conserved Plasmodium protein, unknown function |
| 646921 | 651342 | - | 1.43 | 0.105 | unknown function | PF3D7_1117000 |  | conserved Plasmodium membrane protein, unknown function |
| 1243224 | 1246616 | - | 1.43 | 0.147 | unknown function | PF3D7_1132100 |  | conserved Plasmodium protein, unknown function |
| 686153 | 688873 | + | 1.43 | 0.175 | unknown function | PF3D7_1316500 |  | conserved Plasmodium protein, unknown function |
| 1038172 | 1039680 | - | 1.42 | 0.129 | coding gene | PF3D7_0823500 |  | membrane skeletal protein IMC1-related |
| 982068 | 983404 | + | 1.42 | 0.139 | putative | PF3D7_0721400 |  | P36-like protein homologue, putative |
| 439394 | 443131 | + | 1.42 | 0.145 | putative | PF3D7_0310300 |  | phosphoglycerate mutase, putative |
| 202002 | 203204 | + | 1.42 | 0.065 | putative | PF3D7_0403500 |  | ubiquitin specific protease, putative |
| 1174900 | 1177060 | + | 1.42 | 0.139 | putative | PF3D7_0528500 |  | f-actin capping protein alpha subunit, putative |
| 223731 | 228071 | + | 1.42 | 0.192 | unknown function | PF3D7_0304800 |  | conserved Plasmodium membrane protein, unknown function |
| 284691 | 288023 | + | 1.42 | 0.139 | unknown function | PF3D7_0506800 |  | conserved Plasmodium protein, unknown function |
| 841872 | 842951 | - | 1.41 | 0.095 | coding gene | PF3D7_1320400 |  | type I signal peptidase |
| 2241255 | 2248946 | - | 1.41 | 0.192 | coding gene | PF3D7_1255200 |  | erythrocyte membrane protein 1, PfEMP1 |
| 668562 | 670947 | - | 1.41 | 0.168 | putative | PF3D7_1316000 |  | protein kinase, putative |
| 140049 | 140802 | - | 1.41 | 0.192 | putative | PF3D7_1103100 |  | 60S ribosomal protein P1, putative |
| 478823 | 480946 | - | 1.41 | 0.129 | putative | PF3D7_1112600 |  | DNA helicase, putative |
| 297937 | 301193 | + | 1.41 | 0.081 | putative | PF3D7_0607100 |  | MYND finger protein, putative |
| 798488 | 799918 | + | 1.41 | 0.192 | putative | PF3D7_0919500 |  | sugar transporter, putative |
| 814096 | 818253 | - | 1.41 | 0.129 | unknown function | PF3D7_0717600 |  | conserved Plasmodium protein, unknown function |
| 962155 | 963957 | + | 1.41 | 0.105 | unknown function | PF3D7_1124300 |  | conserved Plasmodium protein, unknown function |
| 1340984 | 1341640 | + | 1.41 | 0.192 | unknown function | PF3D7_1332500 |  | conserved protein, unknown function |
| 2222426 | 2226640 | - | 1.41 | 0.129 | unknown function | PF3D7_1454200 |  | conserved Plasmodium protein, unknown function |
| 2397855 | 2398805 | - | 1.41 | 0.086 | unknown function | PF3D7_1458400 |  | conserved Plasmodium protein, unknown function |
| 126553 | 128375 | - | 1.41 | 0.129 | unknown function | PF3D7_0102800 |  | conserved Plasmodium protein, unknown function |
| 2190467 | 2191754 | - | 1.41 | 0.192 | coding gene | PF3D7_1253700 |  | Rifin |
| 976685 | 977810 | - | 1.41 | 0.192 | putative | PF3D7_0523500 |  | outer arm dynein lc3, putative |
| 644532 | 646388 | + | 1.41 | 0.197 | putative | PF3D7_1216000 |  | seryl-tRNA synthetase, putative |
| 1800550 | 1802298 | + | 1.41 | 0.192 | putative | PF3D7_1242600 |  | protein geranylgeranyltransferase type II, putative |
| 410908 | 416347 | - | 1.41 | 0.197 | unknown function | PF3D7_0309900 |  | conserved Plasmodium protein, unknown function |
| 2085355 | 2086676 | - | 1.41 | 0.189 | unknown function | PF3D7_1251000 |  | conserved Plasmodium protein, unknown function |
