# Supplementary material for: Genome-wide analysis of the human malaria parasite Plasmodium falciparum transcription factor PfNF-YB shows interaction with a CCAAT motif
Source: Oncotarget. 2017 Dec 9;8(69):113987–4001. doi: 10.18632/oncotarget.23053 (PMC5768380; doi:10.18632/oncotarget.23053)
Supplement: Supplementary file 3 [file oncotarget-08-113987-s003.doc]

Supplementary Table 2. Biological functions for PfNF-YB target genes.

| ID | GO Term PlasmoDB |
| --- | --- |
| PF3D7_0405300 | adhesion to host |
| PF3D7_1253700 | antigenic variation |
| PF3D7_0609200 | carbohydrate metabolism |
| PF3D7_1216200 | carbohydrate metabolism |
| PF3D7_1226300 | cell growth process |
| PF3D7_1240000 | cell growth process |
| PF3D7_1342500 | cell motility |
| PF3D7_0919400 | cell redox homeostasis |
| PF3D7_1426900 | cell redox homeostasis |
| PF3D7_0918900 | cell redox homeostasis |
| PF3D7_1440300 | cell redox homeostasis |
| PF3D7_0519300 | cell redox homeostasis |
| PF3D7_0709200 | cell redox homeostasis |
| PF3D7_1367500 | cell redox homeostasis |
| PF3D7_1455800 | cell-cell adhesion |
| PF3D7_1255200 | cell-cell adhesion |
| PF3D7_1105100 | chromatin assembly |
| PF3D7_1224500 | chromatin assembly |
| PF3D7_0528500 | cytoskeleton organization |
| PF3D7_0503400 | cytoskeleton organization |
| PF3D7_1206600 | gene expression regulation |
| PF3D7_1027400 | gene expression regulation |
| PF3D7_1438500 | gene expression regulation |
| [PF3D7_0218300](http://plasmodb.org/plasmo/showRecord.do?name=GeneRecordClasses.GeneRecordClass&source_id=PF3D7_0218300&project_id=PlasmoDB) | gene expression regulation |
| PF3D7_0606500 | gene expression regulation |
| PF3D7_1449300 | gene expression regulation |
| PF3D7_0607100 | gene expression regulation |
| PF3D7_0934400 | gene expression regulation |
| PF3D7_1451400 | gene expression regulation |
| PF3D7_1315600 | lipid metabolism |
| PF3D7_1128400 | lipid metabolism |
| PF3D7_1477800 | lipid metabolism |
| PF3D7_0422000 | lipid metabolism |
| PF3D7_0605900 | lipid metabolism |
| PF3D7_1329400 | nucleic acid metabolism |
| PF3D7_0727800 | nucleic acid metabolism |
| PF3D7_0729500 | nucleic acid metabolism |
| PF3D7_1012400 | nucleic acid metabolism |
| PF3D7_1314200 | nucleic acid metabolism |
| PF3D7_0409600 | nucleic acid metabolism |
| PF3D7_1206100 | nucleic acid metabolism |
| PF3D7_0807900 | nucleic acid metabolism |
| PF3D7_0828200 | nucleic acid metabolism |
| PF3D7_0509600 | nucleic acid metabolism |
| PF3D7_1216000 | nucleic acid metabolism |
| PF3D7_0605600 | nucleic acid metabolism |
| PF3D7_1126900 | nucleic acid metabolism |
| PF3D7_1020400 | nucleic acid metabolism |
| PF3D7_0612600 | nucleic acid metabolism |
| PF3D7_1017900 | proteasomal activity |
| PF3D7_0403500 | proteasomal activity |
| PF3D7_1245300 | proteasomal activity |
| PF3D7_0916500 | proteasomal activity |
| PF3D7_1216300 | protein complex assembly |
| PF3D7_1136800 | protein folding |
| PF3D7_1116800 | protein folding |
| PF3D7_0629200 | protein folding |
| PF3D7_1107500 | protein folding |
| PF3D7_0528700 | protein folding |
| PF3D7_0810500 | protein modification |
| PF3D7_0314400 | protein modification |
| PF3D7_1210900 | protein modification |
| PF3D7_0934800 | protein modification |
| PF3D7_1316000 | protein modification |
| PF3D7_0312400 | protein modification |
| PF3D7_1242600 | protein modification |
| PF3D7_1206200 | protein translation |
| PF3D7_0519400 | protein translation |
| PF3D7_1103100 | protein translation |
| PF3D7_1130200 | protein translation |
| PF3D7_1144000 | protein translation |
| PF3D7_1312400 | protein translation |
| PF3D7_1428600 | protein translation |
| PF3D7_1431000 | protein translation |
| PF3D7_1447300 | protein translation |
| PF3D7_1465900 | protein translation |
| PF3D7_0212200 | protein translation |
| PF3D7_1341300 | protein translation |
| PF3D7_0310300 | protein translation |
| PF3D7_0522500 | protein translation |
| PF3D7_0618300 | protein translation |
| PF3D7_1436800 | proteolysis |
| PF3D7_1320400 | proteolysis |
| PF3D7_1440200 | proteolysis |
| PF3D7_0619400 | regulation of cell cycle |
| PF3D7_1304000 | regulation of cell cycle |
| PF3D7_1112600 | response to DNA damage |
| PF3D7_1012000 | response to DNA damage |
| PF3D7_0605800 | response to DNA damage |
| PF3D7_0709000 | response to drug |
| PF3D7_0308500 | response to stress |
| PF3D7_1124800 | ribosome biogenesis |
| PF3D7_1471000 | ribosome biogenesis |
| PF3D7_1367600 | ribosome biogenesis |
| PF3D7_1360500 | signal transduction |
| PF3D7_0522700 | sulfur metabolism |
| PF3D7_0627500 | sulfur metabolism |
| PF3D7_0807300 | transport |
| PF3D7_1004800 | transport |
| PF3D7_0509000 | transport |
| PF3D7_0919500 | transport |
| PF3D7_1241600 | transport |
| PF3D7_1243500 | transport |
| PF3D7_1250300 | transport |
| PF3D7_1340700 | transport |
| PF3D7_0806800 | transport |
| PF3D7_1447900 | transport |
| PF3D7_1448600 | transport |
| PF3D7_1452500 | transport |
| PF3D7_0316600 | transport |
| PF3D7_0405100 | transport |
| PF3D7_0414700 | transport |
| PF3D7_0926700 | vitamin metabolism |
| PF3D7_0629100 | vitamin metabolism |
| |  | [PF3D7_1116200.1](http://plasmodb.org/plasmo/showRecord.do?name=GeneRecordClasses.GeneRecordClass&source_id=PF3D7_1116200.1&project_id=PlasmoDB) | | --- | --- | | vitamin metabolism |
