# Supplementary material for: Genome-wide analysis of the human malaria parasite Plasmodium falciparum transcription factor PfNF-YB shows interaction with a CCAAT motif
Source: Oncotarget. 2017 Dec 9;8(69):113987–4001. doi: 10.18632/oncotarget.23053 (PMC5768380; doi:10.18632/oncotarget.23053)
Supplement: Supplementary file 4 [file oncotarget-08-113987-s004.docx]

Supplementary Table 4. Common targets of PfNF-YB

| ***Gene ID*** | **target genes of ap2-g** | ***Gene ID P. falciparum**** |
| --- | --- | --- |
| PBANKA_1432400 | perforin like protein 2 (PPLP2) | PF3D7_0406200 |
| PBANKA_0302700 | methyltransferase, putative | PF3D7_1020400 |
| PBANKA_0512000 | DHHC-type zinc finger protein, putative | PF3D7_1322500 |
| PBANKA_0304600 | iron-sulfur assembly protein, putative | PF3D7_0522700 |
| PBANKA_1143400 | NADH-cytochrome b5 reductase, putative | PF3D7_1367500 |
| PBANKA_1454400 | 3-hydroxyisobutyryl-coenzyme A hydrolase, putative | PF3D7_1240000 |
| PBANKA_1108700 | chromosome condensation protein, putative | PF3D7_1304000 |
| PBANKA_1427500 | acyl CoA binding protein, putative | PF3D7_1477800 |
| PBANKA_1456000 | protein geranylgeranyltransferase type II, alpha subunit, putative | PF3D7_1242600 |
| PBANKA_1410900 | translation initiation factor IF-2, putative | PF3D7_1312400 |
| PBANKA_1210400 | ubiquitin-protein ligase e3, putative | PF3D7_1012000 |
| PBANKA_0505000 | dihydrolipoamide acyltransferase, putative | PF3D7_1020800 |
| PBANKA_1329300 | 40S ribosomal protein S3, putative | PF3D7_1465900 |
| PBANKA_0819800 | gamma-glutamylcysteine synthetase (gammaGCS) | PF3D7_0918900 |
| PBANKA_1354100 | Rab GTPase 11b | PF3D7_1340700 |
| PBANKA_1456800 | endosome sorting protein (SNF7 homologue), putative | PF3D7_1243500 |
| PBANKA_0827500 | NAD synthase, putative | PF3D7_0926700 |
| PBANKA_1207800 | metallopeptidase, putative | PF3D7_1009500 |
| PBANKA_0923900 | U6 snRNA-associated Sm-like protein LSm1, putative (LSM1) | PF3D7_0520300 |
| PBANKA_1311700 | multidrug resistance protein 2, putative | PF3D7_1447900 |
| PBANKA_1223800 | vacuolar proton translocating ATPase subunit A, putative | PF3D7_0806800 |
| PBANKA_0836800 | erythrocyte membrane associated protein 1 (EMP1) | PF3D7_1255200 |
| PBANKA_1002900 | ag-1 blood stage membrane protein homologue | PF3D7_0405200 |
| PBANKA_0835600 | cAMP-dependent protein kinase catalytic subunit (PKAc) | PF3D7_0934800 |
| PBANKA_1131900 | serine/threonine protein phosphatase, putative | PF3D7_0314400 |
| PBANKA_0213600 | mRNA (N6-adenosine)-methyltransferase, putative | PF3D7_0729500 |
| PBANKA_1003900 | sexual stage-specific protein precursor, putative | PF3D7_0406200 |
| PBANKA_0308600 | asparagine-tRNA ligase, putative | PF3D7_0509600 |
| PBANKA_1344400 | AMP deaminase, putative | PF3D7_1329400 |
| PBANKA_1439800 | splicing factor 3b subunit, putative (SF3B14) | PF3D7_1224900 |
| PBANKA_1354500 | 60S ribosomal protein L18-2, putative | PF3D7_1341300 |
| PBANKA_0105200 | polypyrimidine tract binding protein, putative | PF3D7_0606500 |
| PBANKA_1302400 | cleavage and polyadenylation specifity factor, putative | PF3D7_1438500 |
| PBANKA_1234200 | 40S ribosomal protein S24, putative | PF3D7_0519400 |
| PBANKA_1324400 | 60S ribosomal protein L27, putative | PF3D7_0618300 |
| PBANKA_0604800 | eukaryotic translation initiation factor 3 subunit 8, putative | PF3D7_1206200 |
| PBANKA_0618800 | DEAD box helicase, putative | PF3D7_1331100 |
| PBANKA_1122000 | coronin binding protein, putative | PF3D7_0623100 |
| PBANKA_0107800 | citrate synthase-like protein, putative | PF3D7_0609200 |
| PBANKA_0502700 | PHF5-like protein, putative | PF3D7_1018500 |
| PBANKA_1034300 | transcription factor with AP2 domain(s), putative | PF3D7_0934400 |
| PBANKA_1010300 | dynein-related AAA-type ATPase, putative | PF3D7_1434500 |
| PBANKA_0934500 | protein tyrosine phosphatase, putative | PF3D7_1331600 |
| PBANKA_0605200 | DNA-directed RNA polymerase III subunit, putative | PF3D7_1206600 |
| PBANKA_0923500 | nuclear preribosomal assembly protein, putative | PF3D7_1124800 |
| PBANKA_1143500 | ribosome biogenesis protein MRT4, putative | PF3D7_1367600 |
| PBANKA_1432500 | DNA-binding chaperone, putative | PF3D7_1216900 |
| PBANKA_0931200 | heat shock protein 101, putative | PF3D7_1116800 |
| PBANKA_1334200 | RNA 3'-terminal phosphate cyclase-like protein, putative | PF3D7_1471000 |
| PBANKA_1313000 | transcription factor IIIb subunit, putative | PF3D7_1449300 |
| **Gene ID** | **target genes of ap2-g2** |  |
| PBANKA_0104400 | nucleoside diphosphate kinase, putative | PF3D7_0605600 |
| PBANKA_1432400 | perforin like protein 2 (PPLP2) | PF3D7_0406200 |
| PBANKA_1411800 | DEAD box helicase, putative | PF3D7_1331100 |
| PBANKA_1143400 | NADH-cytochrome b5 reductase, putative | PF3D7_1367500 |
| PBANKA_1240800 | RAP protein, putative | PF3D7_1470600 |
| PBANKA_1024100 | ATP-dependent RNA helicase, putative | PF3D7_0602100 |
| PBANKA_0903000 | nucleolar preribosomal assembly protein, putative | PF3D7_0818400 |
| PBANKA_1313000 | transcription factor IIIb subunit, putative | PF3D7_1449300 |
| PBANKA_1432500 | DNA-binding chaperone, putative | PF3D7_1216900 |
| PBANKA_1464100 | coronin, putative | PF3D7_0623100 |
| PBANKA_1143500 | ribosome biogenesis protein MRT4, putative | PF3D7_1367600 |
| PBANKA_1355600 | sporozoite protein essential for cell traversal (SPECT) | PF3D7_1342500 |
| PBANKA_1354100 | Rab GTPase 11b | PF3D7_1340700 |
| PBANKA_1344400 | AMP deaminase, putative | PF3D7_1329400 |
| PBANKA_0932900 | glycerol-3-phosphate dehydrogenase, putative | PF3D7_1216200 |
| PBANKA_0209100 | thrombospondin related sporozoite protein (TRSP) | PF3D7_0104000 |
| PBANKA_1456800 | endosome sorting protein (SNF7 homologue), putative | PF3D7_1243500 |
| PBANKA_0605200 | DNA-directed RNA polymerase III subunit, putative | PF3D7_1206600 |
| PBANKA_1006300 | perforin like protein 1 | PF3D7_0408700 |
| PBANKA_1003900 | sexual stage-specific protein precursor, putative | PF3D7_0406200 |
| **Gene ID** | **target genes of PfAP2-l** |  |
| PF3D7_0301800 | Plasmodium exported protein, unknown function |  |
| PF3D7_0503400 | actin-depolymerizing factor |  |
| PF3D7_0532100 | early transcribed membrane protein 5 |  |
| PF3D7_0717600 | conserved Plasmodium protein, unknown function |  |
| PF3D7_0905400 | high molecular weight rhoptry protein 3 |  |
| PF3D7_0934800 | cAMP-dependent protein kinase catalytic subunit |  |
| PF3D7_1105100 | histone H2B |  |
| PF3D7_1206000 | protein phosphatase, putative |  |
| PF3D7_1252100 | conserved Plasmodium protein, unknown function |  |
| PF3D7_1438400 | metacaspase-like protein |  |
| PF3D7_1440200 | stromal-processing peptidase, putative |  |

*FDR score and FC (fold change) see Supplementary table 1.
